# A budding yeast model for human disease mutations in the *EXOSC2* cap subunit of the RNA exosome complex

**DOI:** 10.1261/rna.078618.120

**Published:** 2021-09

**Authors:** Maria C. Sterrett, Liz Enyenihi, Sara W. Leung, Laurie Hess, Sarah E. Strassler, Daniela Farchi, Richard S. Lee, Elise S. Withers, Isaac Kremsky, Richard E. Baker, Munira A. Basrai, Ambro van Hoof, Milo B. Fasken, Anita H. Corbett

**Affiliations:** 1 Biochemistry, Cell and Developmental Biology Graduate Program, Emory University, Atlanta, Georgia 30322, USA; 2 Department of Biology, Emory University, Atlanta, Georgia 30322, USA; 3 Department of Biochemistry, Emory University, Atlanta, Georgia 30322, USA; 4 Loma Linda University School of Medicine, Loma Linda, California 92350, USA; 5 Department of Microbiology and Physiological Systems, University of Massachusetts Medical School, Worcester, Massachusetts 01655, USA; 6 Genetics Branch, Center for Cancer Research, National Cancer Institute, National Institutes of Health, Bethesda, Maryland 20892, USA; 7 Department of Microbiology and Molecular Genetics, University of Texas Health Science Center at Houston, Houston, Texas 77030, USA

**Keywords:** EXOSC2, exosomopathy, RNA decay/processing, RNA exosome, Rrp4

## Abstract

RNA exosomopathies, a growing family of diseases, are linked to missense mutations in genes encoding structural subunits of the evolutionarily conserved, 10-subunit exoribonuclease complex, the RNA exosome. This complex consists of a three-subunit cap, a six-subunit, barrel-shaped core, and a catalytic base subunit. While a number of mutations in RNA exosome genes cause pontocerebellar hypoplasia, mutations in the cap subunit gene *EXOSC2* cause an apparently distinct clinical presentation that has been defined as a novel syndrome SHRF (short stature, hearing loss, retinitis pigmentosa, and distinctive facies). We generated the first in vivo model of the SHRF pathogenic amino acid substitutions using budding yeast by modeling pathogenic *EXOSC2* missense mutations (p.Gly30Val and p.Gly198Asp) in the orthologous *S. cerevisiae* gene *RRP4*. The resulting *rrp4* mutant cells show defects in cell growth and RNA exosome function. Consistent with altered RNA exosome function, we detect significant transcriptomic changes in both coding and noncoding RNAs in *rrp4-G226D* cells that model *EXOSC2* p.Gly198Asp, suggesting defects in nuclear surveillance. Biochemical and genetic analyses suggest that the Rrp4 G226D variant subunit shows impaired interactions with key RNA exosome cofactors that modulate the function of the complex. These results provide the first in vivo evidence that pathogenic missense mutations present in *EXOSC2* impair the function of the RNA exosome. This study also sets the stage to compare exosomopathy models to understand how defects in RNA exosome function underlie distinct pathologies.

## INTRODUCTION

The RNA exosome is an evolutionarily conserved, multisubunit riboexonuclease complex that plays multiple roles in RNA processing and decay. First identified in *Saccharomyces cerevisiae* in a screen for ribosomal RNA processing (*rrp*) mutants (Mitchell et al. 1996, 1997), the RNA exosome is essential in all systems studied thus far (Mitchell et al. 1997; Lorentzen et al. 2007; Hou et al. 2012; Lim et al. 2013; Pefanis et al. 2014). In addition to critical roles in generating mature ribosomal RNA, the RNA exosome processes a variety of small noncoding RNAs (ncRNAs), including small nuclear RNAs (snRNAs), small nucleolar RNAs (snoRNAs), and transfer RNAs (tRNAs) (Allmang et al. 1999; van Hoof et al. 2000; Kilchert et al. 2016a; Fasken et al. 2020). Beyond processing numerous RNAs, the RNA exosome is also required for RNA decay and surveillance, including nuclear degradation of cryptic unstable transcripts (CUTs) in budding yeast that result from pervasive transcription (Wyers et al. 2005; Parker 2012; Schneider et al. 2012) and cytoplasmic RNA turnover of aberrant and nonfunctional mRNAs (Moore and Proudfoot 2009). Therefore, the RNA exosome has many far-reaching roles in vivo that affect nearly every class of RNA (Schneider et al. 2012; Schneider and Tollervey 2013).

**FIGURE 1.**
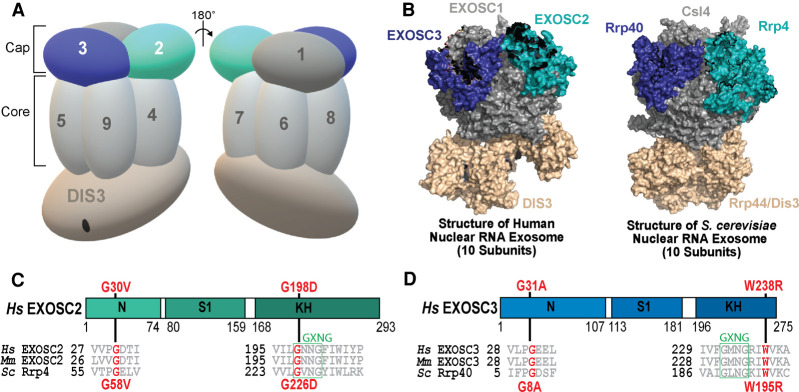
Overview of pathogenic amino acid substitutions in the human cap subunit EXOSC2 of the RNA exosome. (*A*) The RNA exosome is an evolutionary conserved ribonuclease complex composed of nine structural subunits (EXOSC1-9) and one catalytic subunit (DIS3) that form a “cap” and “core” ring-like structure. The three-subunit cap at the *top* of the complex is composed of EXOSC1/Csl4 (Human/*S. cerevisiae*), EXOSC2/Rrp4, and EXOSC3/Rrp40. The six-subunit core is composed of EXOSC4/Rrp41, EXOSC5/Rrp46, EXOSC6/Mtr3, EXOSC7/Rrp42, EXOSC8/Rrp43, and EXOSC9/Rrp45. The DIS3/Dis3/Rrp44 catalytic subunit is located at the *bottom*. Missense mutations in the gene encoding the EXOSC2 cap subunit (teal blue, labeled 2) are linked to a novel syndrome termed SHRF (short stature, hearing loss, retinitis pigmentosa and distinctive facies) (Di Donato et al. 2016). In contrast, missense mutations in the gene encoding the EXOSC3 cap subunit (dark blue, labeled 3) cause PCH1b (pontocerebellar hypoplasia type 1b) (Wan et al. 2012; Biancheri et al. 2013; Eggens et al. 2014; Halevy et al. 2014; Schottmann et al. 2017). (*B*) The structure and organization of the RNA exosome is highly conserved across eukaryotes. A structural model of the human nuclear RNA exosome (*left*) [PDB 6D6R] (Weick et al. 2018) and the *S. cerevisiae* nuclear RNA exosome (*right*) [PDB 6FSZ] (Schuller et al. 2018) are depicted with the cap subunits EXOSC1/Csl4 (Human/*S. cerevisiae*)*,* EXOSC2/Rrp4, EXOSC3/Rrp40, and catalytic subunit DIS3/Dis3/Rrp44 labeled. (*C*,*D*) Domain structures are shown for (*C*) EXOSC2/Rrp4 and (*D*) EXOSC3/Rrp40. Each of these cap subunits is composed of three different domains: an amino-terminal domain, a putative RNA binding S1 domain, and a carboxy-terminal putative RNA binding KH (K homology) domain. The “GxNG” motif identified in the KH domain of both cap subunits is boxed in green. The position of the disease-linked amino acid substitutions in human EXOSC2 and EXOSC3 are depicted *above* the domain structures in red. Sequence alignments of EXOSC2/Rrp4 and EXOSC3/Rrp40 orthologs from *Homo sapiens* (*Hs*), *Mus musculus* (*Mm*), and *S. cerevisiae* (*Sc*) *below* the domain structures show the highly conserved residues altered in disease in red and the conserved sequences flanking these residues in gray. The amino acid substitutions in *S. cerevisiae* Rrp4 generated in this study and those in *S. cerevisiae* Rrp40, described previously (Fasken et al. 2017; Gillespie et al. 2017), that correspond to the disease-linked amino acid substitutions in human EXOSC2 and EXOSC3, are shown *below* the structures in red.

This essential RNA processing/degradation machine is composed of nine structural subunits associated with a catalytic 3′–5′ exo/endoribonuclease subunit (DIS3/DIS3L [human]; Dis3/Rrp44 [budding yeast]) (Mitchell et al. 1997; Makino et al. 2013). As illustrated in Figure 1A, the nine-subunit structural barrel is composed of an upper ring of three S1/KH cap subunits (EXOSC1/2/3; Csl4/Rrp4/Rrp40) and a lower ring of six PH-like subunits (EXOSC4/7/8/9/5/6; Rrp41/Rrp42/Rrp43/Rrp45/Rrp46/Mtr3). Within the nucleus, an additional 3′–5′ exonuclease termed EXOSC10/Rrp6 is also associated with the complex (Briggs et al. 1998; Wasmuth et al. 2014). Structural studies revealed conservation in the structural organization of the RNA exosome (Fig. 1B; Liu et al. 2006; Bonneau et al. 2009; Makino et al. 2013; Wasmuth et al. 2014; Zinder et al. 2016), suggesting evolutionary conservation not just of subunit sequence but of overall complex structure and organization.

As described above, a feature of the RNA exosome is the ability to both process and degrade numerous RNA targets. The specificity for this broad set of target transcripts is achieved, at least in part, through RNA exosome cofactors that associate with the complex via interactions with multiple subunits (Zinder and Lima 2017; Fasken et al. 2020). These cofactors are localized to both the nucleus and the cytoplasm, raising the possibility that these interactions facilitate targeting of distinct target transcripts in different cellular compartments. Nuclear RNA exosome cofactors have been extensively characterized in the budding yeast system, including the Rrp6 obligate binding partner Rrp47, the essential RNA helicase Mtr4, and Mpp6 (de la Cruz et al. 1998; LaCava et al. 2005; Schneider and Tollervey 2013; Zinder and Lima 2017). Structural studies of both the budding yeast and mammalian RNA exosome suggest Mpp6/MPH6, Rrp6/EXOSC10, and Rrp47/C1D can form composite sites that facilitate interactions between the complex and other cofactors, including Mtr4/MTR4/MTREX (Schuch et al. 2014; Falk et al. 2017; Wasmuth et al. 2017; Schuller et al. 2018; Weick et al. 2018). Mtr4/MTR4/MTREX aids the RNA exosome in targeting and processing target RNA, such as the 5.8S rRNA precursor (7S rRNA), and can act as part of the budding yeast TRAMP (Trf4/5-Air1/2-Mtr4 Polyadenylation) complex or the mammalian NEXT (Nuclear Exosome Targeting) complex, both of which facilitate nuclear RNA surveillance and quality control of ncRNA by the RNA exosome (de la Cruz et al. 1998; LaCava et al. 2005; Vaňáčová et al. 2005; Houseley and Tollervey 2008, 2009; Lubas et al. 2011; Stuparevic et al. 2013; Schuch et al. 2014; Rodríguez-Galán et al. 2015; Kilchert et al. 2016b; Falk et al. 2017; Zinder and Lima 2017). Thus, interactions with these nuclear cofactors influence the function of the RNA exosome in vivo.

Recent studies have linked missense mutations in *EXOSC* genes encoding the structural subunits of the RNA exosome to various human pathologies, which comprise a growing family of diseases termed “RNA exosomopathies” (Wan et al. 2012; Biancheri et al. 2013; Boczonadi et al. 2014; Eggens et al. 2014; Di Donato et al. 2016; Schottmann et al. 2017; Burns et al. 2018; Fasken et al. 2020; Slavotinek et al. 2020). Intriguingly, these single amino acid substitutions often occur in highly conserved domains of the RNA exosome subunits. Mutations in the cap subunit gene *EXOSC3* and the core subunit gene *EXOSC8* cause forms of pontocerebellar hypoplasia (PCH1b and PCH1c, respectively), neurological disorders characterized by atrophy of the pons and cerebellum (Wan et al. 2012; Biancheri et al. 2013; Boczonadi et al. 2014; Eggens et al. 2014; Schottmann et al. 2017; Morton et al. 2018), while mutations in the core subunit genes *EXOSC5* and *EXOSC9* have been linked to similar neurological defects including cerebellar degeneration, neuronopathy and neurodevelopmental delays (Burns et al. 2017, 2018; Slavotinek et al. 2020). In contrast to the other exosomopathy mutations described thus far, missense mutations in the cap subunit gene *EXOSC2* have been linked to a novel, complex syndrome characterized by retinitis pigmentosa, progressive hearing loss, premature aging, short stature, mild intellectual disability and distinctive gestalt (Di Donato et al. 2016), later named SHRF (short stature, hearing loss, retinitis pigmentosa and distinctive facies) (OMIM #617763) (Yang et al. 2019). While SHRF patients do show some cerebellar atrophy (Di Donato et al. 2016), the disease phenotype is distinct from PCH as well as the other neurological deficits observed in patients with other exosomopathies, suggesting a unique molecular pathology linked to *EXSOC2* mutations.

Whole exome sequencing of the three identified SHRF patients, representing two related patients and one unrelated patient, identified missense mutations in the *EXOSC2* gene that alter conserved amino acids in this cap subunit, shown in Figure 1C (Di Donato et al. 2016). The two related patients have a homozygous missense mutation *EXOSC2* p.Gly30Val (G30V) in the amino-terminal domain of EXOSC2 (Di Donato et al. 2016). The other patient carries compound heterozygous missense mutations *EXOSC2* p.Gly30Val and *EXOSC2* p.Gly198Asp (G30V/G198D), with the G198D missense mutation located within the K-homology (KH) RNA binding domain (Di Donato et al. 2016; Yang et al. 2019). These amino acid substitutions occur in highly conserved residues of EXOSC2, which are conserved across EXOSC2/Rrp4 orthologs from different eukaryotic species and conserved between EXOSC2 and the EXOSC3/Rrp40 cap subunits of the eukaryotic RNA exosome (Fig. 1D; Supplemental Fig. S1). Notably, EXOSC2 Gly30 and EXOSC3 Gly31, an amino acid that is substituted in PCH1b patients (Wan et al. 2012), are conserved and in the same position in the two cap subunits, falling within a conserved “VxPG” consensus sequence (Supplemental Fig. S1). EXOSC2 Gly198 and EXOSC3 Trp238, another amino acid that is substituted in PCH1b patients (Wan et al. 2012), lie in the KH domains of the two cap subunits, falling within or adjacent to a conserved “GxNG” motif (Fig. 1D). The “GxNG” motif is unique to the KH domain of these RNA exosome cap subunits and is predicted to play a structural role (Oddone et al. 2007). However, when EXOSC2 Gly30, EXOSC2 Gly198, and EXOSC3 Gly31, EXOSC3 Trp238 are substituted in compound heterozygous variants, they give rise to distinct disease phenotypes, suggesting that similar missense mutations in *EXOSC2* and *EXOSC3* have different mechanistic effects on the function of the RNA exosome in vivo. Therefore, to better understand the molecular pathology of these exosomopathies, including SHRF, it is necessary to investigate the molecular and functional consequences of pathogenic amino acid substitutions that underlie each disease.

A previous study provided some important insights into how the *EXOSC2* mutations that cause SHRF could contribute to pathology (Yang et al. 2019). This study used several different approaches, including using patient B-lymphoblasts, in vitro cell culture and a *Drosophila melanogaster* model depleted for the fly EXOSC2/Rrp4 ortholog. Taken together, results from this study suggest that EXOSC2 dysfunction could compromise downstream molecular pathways, including neurodevelopment and autophagy (Yang et al. 2019). While informative in probing the molecular pathology that may underlie the SHRF syndrome, a limitation of this study is that the authors did not examine known targets of the RNA exosome nor did they examine the in vivo consequences of the SHRF-linked EXOSC2 variants within a whole organism. Given that this diverse class of RNA exosomopathies arises from amino acid substitutions in structural subunits of a singular complex, assessing defects in RNA exosome function in vivo is critical for a holistic understanding of the molecular and functional consequences underlying each disease phenotype. Previous studies have assessed the functional and molecular consequences of exosomopathy-linked *EXOSC3* and *EXOSC5* mutations in vivo using yeast and fly genetic model systems (Fasken et al. 2017; Gillespie et al. 2017; de Amorim 2020; Morton et al. 2020; Slavotinek et al. 2020). Utilizing a genetic model system to explore the consequences of the specific amino acid changes that occur in SHRF can provide insight into how RNA exosome function is altered in disease.

To explore the functional consequences of the amino acid substitutions in EXOSC2 that occur in SHRF, we took advantage of the budding yeast model system. We generated variants of the *S. cerevisiae* EXOSC2 ortholog, Rrp4, that model the pathogenic amino acid substitutions and examined their function in budding yeast. Our results show that the yeast Rrp4 G58V variant, corresponding to the EXOSC2 G30V variant, is not able to replace the function of the essential *RRP4* gene. In contrast, cells that express the Rrp4 G226D variant, corresponding to the EXOSC2 G198D variant, show impaired cell growth and defects in RNA exosome function, revealing that this Rrp4 G226D variant is functional but impaired. Based on RNA-seq analysis, the *rrp4-G226D* cells show broad transcriptomic changes with defects particularly in nuclear surveillance by the RNA exosome. Genetic and biochemical studies demonstrate that the rrp4-G226D variant exhibits impaired interactions with RNA exosome cofactor mutants, likely suggesting defects in association with Mtr4. Combined, these results suggest that amino acid changes in Rrp4 that model those in EXOSC2 impair the overall function of the RNA exosome, potentially through impairment of interactions with the exosome cofactor Mtr4.

## RESULTS

### EXOSC2 amino acid substitutions linked to SHRF are located in conserved domains

**FIGURE 2.**
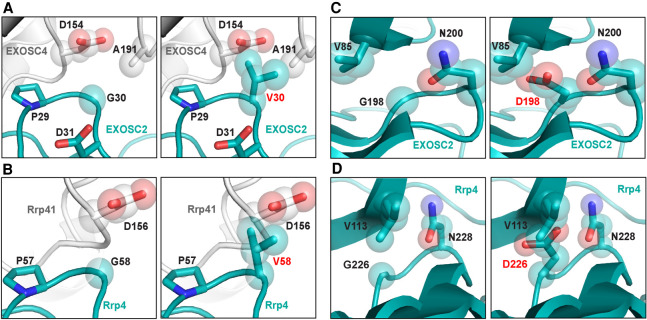
Modeling pathogenic amino acid substitutions in Human EXOSC2 and *S. cerevisiae* Rrp4. (*A*) Structural modeling of the human EXOSC2 p.Gly30Val (G30V) amino acid substitution identified in patients with SHRF syndrome in the human RNA exosome. Zoomed-in representations of the interface between EXOSC2 (teal blue) and EXOSC4 (light gray) modeling the native EXOSC2 Gly30 (G30) residue (*left*) or the pathogenic EXOSC2 Val30 (V30) residue (*right*) are depicted. The EXOSC2 Gly30 residue is located in the amino-terminal domain of EXOSC2, near the interface of EXOSC2 with the core subunit, EXOSC4. (*B*) Structural modeling of budding yeast Rrp4 Gly58Val (G58V) amino acid change, corresponding to EXOSC2 p.Gly30Val amino acid change, in the budding yeast exosome. Zoomed-in representations of the interface between Rrp4 (teal blue) and the budding yeast EXOSC4 ortholog, Rrp41 (light gray), modeling the native Rrp4 Gly58 (G58) residue (*left*) or the modeled pathogenic Rrp4 Val58 (V58) residue (*right*) are shown. The Rrp4 Gly58 residue is conserved between human and yeast and, similarly to EXOSC2 Gly30, is located in the amino-terminal domain of Rrp4, near the interface of Rrp4 with the core subunit, Rrp41. (*C*) Structural modeling of the EXOSC2 p.Gly198Asp (G198D) amino acid substitution identified in patients with SHRF syndrome in the human RNA exosome. Zoomed-in representations of EXOSC2 modeling the native EXOSC2 Gly198 (G198) residue (*left*) or the pathogenic EXOSC2 Asp198 (D198) residue (*right*) are shown. The EXOSC2 Gly198 residue is located in the KH-domain of EXOSC2 within a dense region of the protein, surrounded by four β-sheets. (*D*) Structural modeling of the budding yeast Rrp4 Gly226Asp (G226D) amino acid change, corresponding to the EXOSC2 p.Gly198Asp amino acid change, in the budding yeast RNA exosome. Zoomed-in representations of Rrp4 modeling the native Rrp4 Gly226 (G226) residue (*left*) or the modeled pathogenic Rrp4 Asp226 (D226) residue (*right*) are shown. The Rrp4 Gly226 residue, which is conserved between human and yeast, is located in the KH-domain of Rrp4 within a dense region of the protein, surrounded by four β-sheets. Structural modeling in *A* and *C* was performed with the human RNA exosome structure (PDB 6D6R) (Weick et al. 2018) and in *B* and *D* with the yeast RNA exosome structure (PDB 6FSZ) (Schuller et al. 2018) using PyMOL (The PyMOL Molecular Graphics System, Version 2.0 Schrödinger, LLC).

To explore how EXOSC2 G30V and EXOSC2 G198D variants could alter the structure of the EXOSC2 protein or the RNA exosome complex (Fig. 1C), we modeled these EXOSC2 amino acid substitutions using recent structures of the human RNA exosome (PDB 6D6R [Fig. 2A,C; Weick et al. 2018]) and the *S. cerevisiae* RNA exosome (PDB 6FSZ [Fig. 2B,D; Schuller et al. 2018]). Structural modeling shows that the EXOSC2 Gly30 residue is positioned at the interface with the core subunit EXOSC4 toward the exterior of the complex in a region with little disorder (Fig. 2A). The EXOSC2 Gly30 residue lies in a β-turn next to a highly conserved proline (Pro29), which is likely essential for the region to have the flexibility needed to make the sharp turn observed in the structure. EXOSC2 Gly30 is also adjacent to an aspartic acid (Asp31), which forms a salt bridge with Arg232 of EXOSC4, likely stabilizing the interaction between the two subunits (Supplemental Fig. S2A). An amino acid substitution that alters the glycine at position 30 is predicted to alter the β-turn and position the Asp31 residue away from Arg232 such that the salt bridge would be disrupted. In addition, the EXOSC2 G30V substitution introduces a valine, which is significantly larger than glycine and appears to clash with residues Asp154 and Ala191 in EXOSC4 (Fig. 2A), suggesting this substitution could negatively impact the interactions between the cap EXOSC2 and core EXOSC4 subunit. In the budding yeast structure, Rrp4 Gly58, corresponding to EXOSC2 Gly30 (Fig. 1C), is located at the interface with Rrp41, the budding yeast EXOSC4 ortholog, in a β-turn adjacent to a highly conserved proline (Pro57) in Rrp4 (Fig. 2B), mirroring the human structural model (Fig. 2A). Rrp4 Gly58 is located next to a glutamic acid (Glu59; Supplemental Fig. S2B) in Rrp4 that forms a salt bridge with Arg233 in Rrp41, similar to the EXSOC2–EXOSC4 interface (Supplemental Fig. S2A). Similar to the human structure, in the yeast exosome structure, the Rrp4 Gly58 residue is also predicted to be essential for the flexibility of the region, facilitating the β-turn, and thus stabilizing the Rrp4–Rrp41 interface (Supplemental Fig. S2B). Similar to the EXOSC2 G30V substitution, substitution of Gly58 in Rrp4 most likely disrupts this β-turn, disrupting the salt bridge and destabilizing the Rrp4–Rrp41 interface.

EXOSC2 Gly198 is positioned in a dense region of the subunit, surrounded by four β sheets (Fig. 2C). The EXOSC2 G198D substitution introduces the large aspartic acid residue which appears to clash with neighboring residues Val85 and Asn200 (Fig. 2C) and alter EXOSC2 conformation. In addition, the EXOSC2 G198D substitution introduces a polar aspartic acid residue in place of glycine with an electronegative oxygen that would undergo repulsion with the oxygen of Asn200, making the native structure depicted in Figure 2C unlikely for this variant. Structural modeling of the Rrp4 Gly226 residue, corresponding to EXOSC2 Gly198 (Fig. 1C), shows that, like Gly198 in EXOSC2, Gly226 residue is positioned in a dense region of Rrp4, surrounded by four β sheets (Fig. 2D). The residues neighboring Rrp4 Gly226, Val113, and Asn228, are highly conserved and correspond to EXOSC2 Val85 and Asn200, suggesting that the budding yeast Rrp4 G226D substitution can accurately model the structural changes predicted for the human EXOSC2 G198D substitution.

The online server mCSM-PPI2 was used to calculate the change in Gibbs free energy (ΔΔG) to predict the effect of the EXOSC2 amino acid substitutions and corresponding Rrp4 amino acid substitutions on protein–protein interactions. Consistent with observations from structural modeling, the software predicts destabilizing changes in the affinity of the protein–protein interactions for both EXOSC2 G30V (ΔΔG = −1.012 Kcal/mol) and EXOSC2 G198D (ΔΔG = −0.509 Kcal/mol). The EXOSC2 G198D substitution is also predicted to reduce protein stability (Score = 1.000 Polymorphism Phenotyping v2). These predictions are consistent with previous work showing that EXOSC2 G198D has reduced stability compared to wild-type EXOSC2 (Yang et al. 2019). Furthermore, both substitutions are strongly predicted to have deleterious effects on EXOSC2 function (G30V score −7.938 and G198D score −6.35 calculated by PROVEAN; G30V score 91, G198D score 94 calculated by SNAP-2). Both Rrp4 G58V (which models EXOSC2 G30V) and Rrp4 G226D (which models EXOSC2 G198D) are predicted to decrease protein stability (Score = 1.000 Polymorphism Phenotyping v2) as well as to have deleterious effects on function (G58V score −8.981 and G226D score −6.517 calculated by PROVEAN). Rrp4 G58V is likely to alter the native protein (score 63 calculated by SNAP2), though to a slightly lower degree than calculated for the human EXOSC2 G30V variant. However, Rrp4 G226D likely results in a change to the native protein (score 92 by SNAP2), mirroring the strong effect predicted for the human EXOSC2 G198D variant. In conclusion, these in silico predictions (summarized in Supplemental Table S3) suggest that the pathogenic amino acid substitutions have molecular consequences that could affect RNA exosome function in both humans and budding yeast.

### *Saccharomyces cerevisiae* Rrp4 variants that model the pathogenic EXOSC2 variants impair RNA exosome function

**FIGURE 3.**
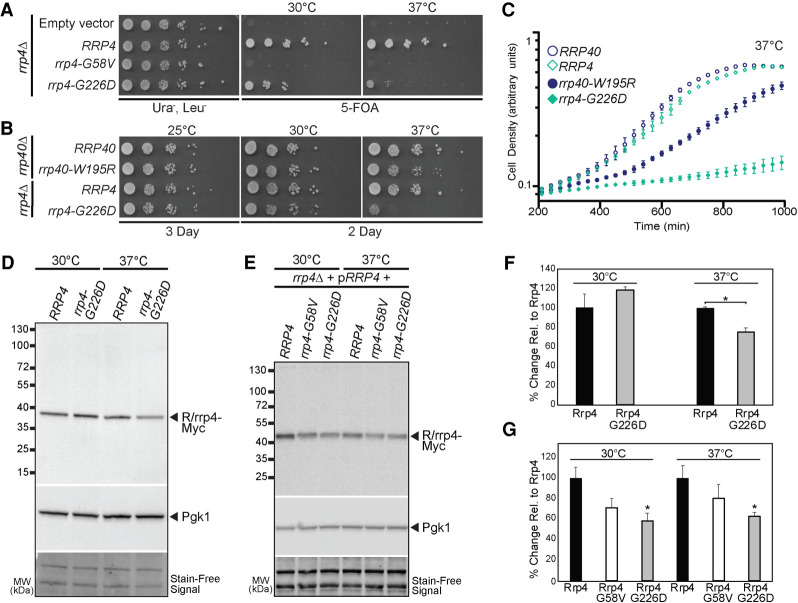
*S. cerevisiae* Rrp4 variants that model EXOSC2 variants identified in patients show impaired function. *S. cerevisiae* cell expressing Rrp4 variants that model pathogenic amino acid changes found in EXOSC2 were generated as described in Materials and Methods. (*A*) Although cell growth is comparable for all mutants that contain a wild-type *RRP4* maintenance plasmid (Ura^−^ Leu^−^), *rrp4-G58V* mutant cells are not viable on plates containing 5-FOA where the maintenance plasmid is not present. The *rrp4-G226D* cells show temperature-sensitive growth on 5-FOA relative to control *RRP4* cells. The cells were grown at the indicated temperatures. (*B*,*C*) The *rrp4-G226D* cells exhibit profoundly impaired growth compared to control *RRP4* cells at 37°C as assessed by (*B*) serial dilution growth assay on plates or (*C*) growth in liquid media. (*B*) The *rrp4*Δ cells expressing only *RRP4* or *rrp4-G226D* and *rrp40*Δ cells expressing only *RRP40* or *rrp40-W195R* were serially diluted, spotted onto solid media grown at the indicated temperatures or (*C*) grown in liquid media at 37°C with optical density measurement used to assess cell density over time. The growth of *rrp40-W195R* cells, previously reported to be moderately impaired at 37°C (Fasken et al. 2017; Gillespie et al. 2017), was included as a comparative control. (*D*) The steady-state level of the Rrp4 G226D protein variant is modestly decreased at 37°C. Lysates of *rrp4*Δ cells solely expressing Myc-tagged wild-type Rrp4 or rrp4-G226D grown at 30°C or 37°C were analyzed by immunoblotting with an anti-Myc antibody to detect Rrp4-Myc and an anti-Pgk1 antibody to detect 3-phosphoglycerate kinase (Pgk1) as a loading control. (*E*) The Rrp4-G58V protein variant is expressed and the steady-state level of the Rrp4 G226D protein variant is decreased in cells coexpressing wild-type Rrp4. Lysates of *rrp4*Δ cells coexpressing untagged wild-type Rrp4 and Myc-tagged wild-type Rrp4, Rrp4 G58V, or Rrp4 G226D grown at 30°C were analyzed by immunoblotting with an anti-Myc antibody to detect Rrp4-Myc and anti-Pgk1 antibody to detect 3-phosphoglycerate kinase (Pgk1) as loading control. (*F*) Quantitation of the percentage of Rrp4 or Rrp4 G226D protein detected in lysates of *rrp4*Δ cells solely expressing Myc-tagged Rrp4 or Rrp4 G226D grown at 30°C or 37°C. Graph shows the mean percentage of Rrp4-Myc protein from three independent experiments (*n* = 3). Error bars represent standard error of mean. Statistical significance is denoted by asterisk (*P-value ≤0.05). (*G*) Quantitation of the percentage of Rrp4, Rrp-G58V and Rrp4-G226D protein detected in lysates of *rrp4*Δ cells expressing Myc-tagged Rrp4 or Rrp4 variants grown at 30°C or 37°C. Graph shows the mean percentage of Rrp4-Myc from three independent experiments (*n* = 3). Error bars represent standard error of mean. Statistical significance is denoted by asterisk (*P-value ≤0.05). Quantitation of immunoblots in *F* and *G* was performed as described in Materials and Methods.

To assess the in vivo consequences of the pathogenic amino acid substitutions in EXOSC2, G30V, and G198D, we generated the corresponding amino acid changes in the *S. cerevisiae* ortholog Rrp4, G58V, and G226D (Fig. 1C). As all core RNA exosome subunits genes are essential in budding yeast (Allmang et al. 1999), we first assessed whether these *rrp4* gene mutants can replace the essential *RRP4* gene. To facilitate comparison of different *rrp4* mutants, we used a plasmid shuffle assay in which cells deleted for the genomic copy of *RRP4* are transformed with plasmids containing mutant alleles (See Materials and Methods). This approach ensures that the genetic background for all mutants compared to one another is identical (Sikorski and Hieter 1989). In this plasmid shuffle assay, *rrp4*Δ cells containing a *RRP4* maintenance plasmid and either *rrp4-G58V* or *rrp4-G226D* plasmid were serially diluted and spotted onto 5-FOA plates to select for cells that harbor the *rrp4* mutant as the sole copy of *RRP4* (Fig. 3A). The *rrp4-G58V* mutant cells are not viable at any temperature tested, whereas the *rrp4-G226D* cells exhibit impaired growth at 37°C as compared to control *RRP4* cells (Fig. 3A). Control cells expressing wild-type *RRP4* grow at all temperatures as expected. The impaired growth of *rrp4-G226D* mutant cells was further analyzed by serial dilution and spotting on solid minimal media (Fig. 3B) and in a liquid media growth assay (Fig. 3C). On solid media and in liquid culture, the *rrp4-G226D* cells show impaired growth at 37°C compared to control *RRP4* cells (Fig. 3B,C). For comparison, we also assessed the growth of the previously characterized *rrp40-W195R* mutant cells (Fasken et al. 2017; Gillespie et al. 2017), which solely express the *rrp40-W195R* mutant corresponding to the *EXOSC3-W238R* mutant linked to PCH1b (Wan et al. 2012; Biancheri et al. 2013; Eggens et al. 2014; Halevy et al. 2014; Schottmann et al. 2017). The *rrp4-G226D* cells exhibit a more profound growth defect than *rrp40-W195R* cells at 37°C as determined by comparing each mutant to the corresponding wild-type control (Fig. 3B,C).

The growth defects associated with the *rrp4* mutant cells could be due to a decrease in the level of the essential Rrp4 protein. To explore this possibility, we examined the expression of Myc-tagged wild-type Rrp4 and Rrp4 variants by immunoblotting and quantitated the changes in steady-state level of Rrp4 G226D-Myc and Rrp4 G58V-Myc compared to wild-type control (Fig. 3D–F). We first examined the steady-state levels of Myc-tagged Rrp4 G226D when expressed as the sole copy of the Rrp4 protein in *rrp4*Δ cells grown at either 30°C or 37°C. Immunoblotting reveals that the steady-state level of Rrp4 G226D is comparable to wild-type Rrp4 at 30°C; however, at 37°C, the level of Rrp4 G226D is decreased to ∼75% of that of wild-type Rrp4 (Fig. 3D). As Rrp4 G58V does not support cell viability, we could not examine the expression of this variant as the sole copy of Rrp4 in cells. Thus, we examined the expression of Myc-tagged Rrp4, Rrp4 G58V, and Rrp4 G226D in the presence of *RRP4*. Under these conditions, where an untagged copy of Rrp4 is present, the steady-state level of Rrp4 G58V-Myc is decreased to ∼68% that of wild-type Rrp4 and Rrp4 G226D-Myc is decreased to ∼51% that of the wild-type Rrp4 at both 30°C and 37°C (Fig. 3E). These data show that the level of Rrp4 G58V is not decreased more than Rrp4 G226D in the presence of a wild-type copy of Rrp4. Quantitation of results from these studies are shown in Figure 3F and G. The Rrp4 variants show a decrease in steady-state levels in the presence of wild-type Rrp4, suggesting cells can discriminate between wild-type and variant RNA exosome subunits.

### The Rrp4 G226D variant can associate with the RNA exosome complex in vivo in the absence of competing wild-type Rrp4

**FIGURE 4.**
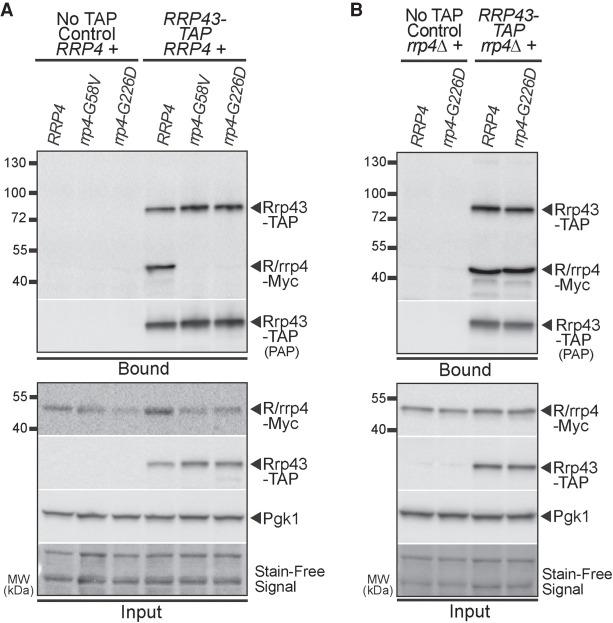
The Rrp4 G58V and Rrp4 G226D variants do not associate with the RNA exosome complex in the presence of wild-type Rrp4, but the Rrp4 G226D variant can associate with the RNA exosome complex. (*A*) The Rrp4 G226D and Rrp4 G58V variants do not associate with the RNA exosome core subunit Rrp43 in the presence of a wild-type copy of Rrp4. TAP-tagged Rrp43 was immunoprecipitated from *RRP43-TAP* cells expressing Myc-tagged Rrp4*,* Rrp4 G58V, or Rrp4 G226D in the presence of wild-type Rrp4 grown at 30°C using IgG Sepharose beads and analyzed by immunoblotting. As a control, immunoprecipitations were also performed from untagged *RRP43* cells (No TAP Control) expressing Myc-tagged Rrp4 proteins. The bound/input level of Rrp4-Myc was detected with an anti-Myc antibody and bound/input level of Rrp43-TAP was detected with a peroxidase antiperoxidase (PAP) antibody. Bound Rrp43-TAP was also detected by the anti-Myc antibody as the Protein A moiety of the TAP tag binds to antibody. The input level of 3-phosphoglycerate kinase (Pgk1) was detected with an anti-Pgk1 antibody as a loading control. The stain-free signal from input protein is also included as a loading control. (*B*) The Rrp4 G226D variant associates with the RNA exosome core subunit Rrp43 at a level similar to wild-type Rrp4 when the variant is the sole form of Rrp4. TAP-tagged Rrp43 was immunoprecipitated from *RRP43-TAP rrp4*Δ cells expressing either Myc-tagged wild-type Rrp4 or Rrp4 G226D, which were grown at 30°C using IgG Sepharose beads and analyzed by immunoblotting. As a control, immunoprecipitations were also performed from untagged *RRP43 rrp4*Δ cells (No TAP Control) expressing Myc-tagged Rrp4 proteins. The bound/input level of Rrp4-Myc was assessed with an anti-Myc antibody and bound/input level of Rrp43-TAP was detected with a peroxidase anti-peroxidase (PAP) antibody. Bound Rrp43-TAP was also detected by the anti-Myc antibody as the Protein A moiety of the TAP tag binds to antibody. The input level of 3-phosphoglycerate kinase (Pgk1) was detected with an anti-Pgk1 antibody as a loading control. The immunoblots are representative of triplicate experiments; coimmunoprecipitations were performed as described in Materials and Methods.

The amino acid substitutions in the Rrp4 variants could decrease association of Rrp4 with the other cap and/or core subunits of the RNA exosome, as reported for human EXOSC2 G30V and EXOSC2 G198D (Yang et al. 2019). To initially examine the association of Rrp4 variants with the RNA exosome, we performed coimmunoprecipitations using *RRP43-TAP* cells that contain the endogenous *RRP4* gene and express a carboxy-terminally tandem affinity purification (TAP)-tagged Rrp43 core subunit from the endogenous *RRP43* locus. We coexpressed Rrp4-Myc, Rrp4 G58V-Myc, or Rrp4 G226D-Myc in these *RRP43-TAP* cells. The Rrp43-TAP protein was immunoprecipitated and association of the Myc-tagged Rrp4 variants was assayed by immunoblotting (Fig. 4A). Under these conditions where an endogenous, wild-type copy of *RRP4* is present, we do not detect association of Rrp4 G58V-Myc or Rrp4 G226D-Myc with Rrp43-TAP, whereas we do detect association of the wild-type Rrp4-Myc with Rrp43-TAP (Fig. 4A).

To further investigate association of Rrp4 G226D with the RNA exosome complex, we performed the same coimmunoprecipitation experiment in the absence of endogenous *RRP4* using *RRP43-TAP rrp4*Δ cells that express Rrp4-Myc or Rrp4 G226D-Myc. We could not express Rrp4 G58V-Myc in these cells as Rrp4 G58V does not support viability (Fig. 3A). The Rrp43-TAP protein was immunoprecipitated and association of Rrp4-Myc or Rrp4 G226D-Myc was assayed by immunoblotting (Fig. 4B). Under these conditions where Rrp4 G226D-Myc is the sole copy of the essential cap subunit, we detect association with the RNA exosome complex at levels comparable to wild-type Rrp4-Myc (Fig. 4B). These data suggest that Rrp4 G226D can associate with the RNA exosome complex when it is the sole copy of the cap subunit; however, in the presence of endogenous *RRP4*, the wild-type copy of Rrp4 can out compete pathogenic Rrp4 variants for incorporation into the RNA exosome complex.

### The Rrp4 G226D variant impairs RNA exosome function

**FIGURE 5.**
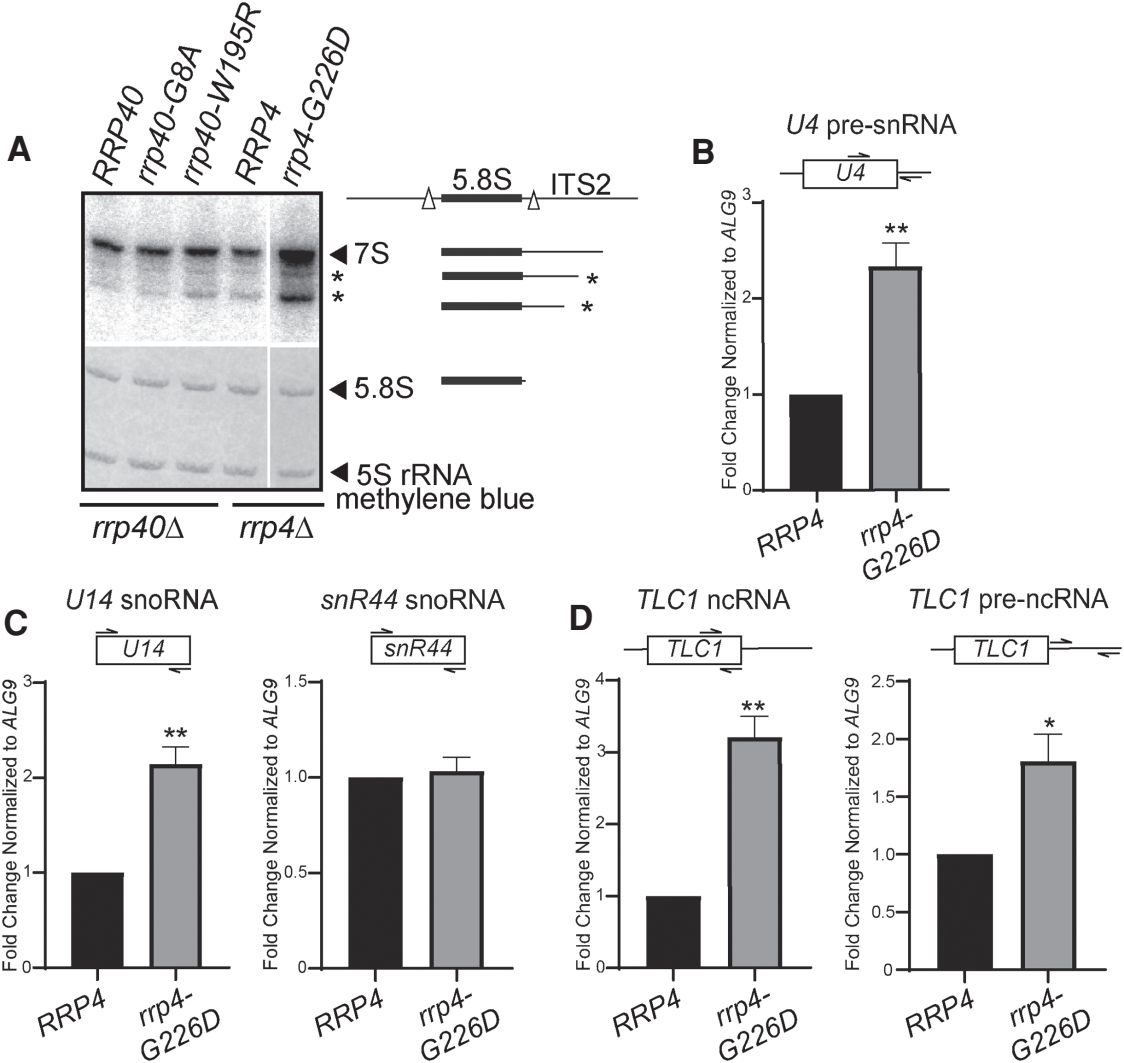
The *rrp4-G226D* variant cells show elevated levels of some but not all RNA exosome target transcripts. (*A*) The *rrp4-G226D* cells exhibit greater accumulation of 7S pre-RNA compared to *RRP4* and *rrp40-W195R* cells grown at 37°C. Total RNA from *RRP40*, *rrp40-G8A*, *rrp40-W195R*, *RRP4*, and *rrp4-G226D* cells grown at 37°C was analyzed by northern blotting with a 5.8S-ITS2 probe to detect 7S pre-rRNA. Mature 5.8S rRNA and 5S rRNA were detected by methylene blue staining as a loading control. The 7S pre-rRNA is normally processed to mature 5.8S rRNA by 3′–5′ decay of the internal transcribed spacer 2 (ITS2) via the nuclear RNA exosome (Mitchell et al. 1996; Allmang et al. 1999). All lanes are imaged from the same northern blot with a gap in the loading indicated by the white line. The simplified schematics to the *right* illustrate the processing steps of 7S rRNA precursor following endonucleolytic cleavage from the larger 27S precursor (indicated by white triangles). (*B*) The *rrp4-G226D* cells show an elevated steady-state level of 3′-extended pre-*U4* snRNA relative to *RRP4* cells at 37°C. (*C*) The *rrp4-G226D* cells exhibit an increased steady-state level of *U14* (*snR128*) snoRNA but not *snR44* snoRNA relative to *RRP4* cells at 37°C. (*D*) The *rrp4-G226D* cells show an elevated steady-state level of mature and extended precursor *TLC1* telomerase component ncRNA relative to *RRP4* cells at 37°C. In *B*–*D*, total RNA was isolated from cells grown at 37°C and transcript levels were measured by RT-qPCR using gene specific primers, normalized relative to *RRP4,* and graphed as described in Materials and Methods. Gene specific primer sequences are summarized in Supplemental Table S2 and their locations within the transcript are graphically represented by the cartoons *above* each bar graph. Within the cartoon transcript, the box represents the body of the mature transcript. Error bars represent standard error of the mean from three biological replicates. Statistical significance of the RNA levels in *rrp4-G226D* cells relative to *RRP4* cells is denoted by an asterisk (*P-value ≤0.05; **P-value ≤0.01).

To assess the function of the RNA exosome in *rrp4-G226D* cells, we examined the steady-state levels of several well-defined RNA exosome target transcripts. The RNA exosome has a critical role in ribosomal RNA (rRNA) processing, specifically processing 7S pre-rRNA into mature 5.8S rRNA (Mitchell et al. 1996; Allmang et al. 1999). We analyzed the processing of 5.8S rRNA in *rrp4-G226D* cells using northern blotting. We also compared 5.8S rRNA processing in *rrp4-G226D* cells to yeast cells modeling *EXOSC3* PCH1b mutations, *rrp40-G8A* and *rrp40-W195R* (Fasken et al. 2017; Gillespie et al. 2017). As shown in Figure 5A, *rrp4-G226D* cells accumulate 7S pre-rRNA, a precursor of mature 5.8S rRNA. In addition, several intermediate precursors of 5.8S rRNA, indicated by asterisks, accumulate in *rrp4-G226D* cells. Despite the accumulation of precursors, the level of mature 5.8S rRNA does not appear to differ in *rrp4-G226D* cells compared to control *RRP4* cells. The accumulation of 7S pre-rRNA and other 5.8S rRNA precursors in *rrp4-G226D* cells is greater than that detected in *rrp40-W195R* cells (Fig. 5A; Supplemental Fig. S3), which have documented accumulation of this rRNA precursor (Gillespie et al. 2017).

We next analyzed the steady-state levels of several RNA exosome target transcripts in *rrp4-G226D* cells using quantitative RT-PCR (Allmang et al. 1999). The *rrp4-G226D* cells exhibit a significant increase in the level of 3′-extended *U4* pre-snRNA compared to *RRP4* control cells (Fig. 5B). The *rrp4-G226D* cells exhibit a significant increase in the level of *U14* box C/D snoRNA, whereas they show no significant difference in the level of the *snR44* box H/ACA snoRNA (Fig. 5C). We also measured the steady-state level of the telomerase RNA *TLC1,* which is processed by the RNA exosome in a pathway similar to pre-snRNA processing (Coy et al. 2013). The *rrp4-G226D* cells exhibit a significant increase in the steady-state level of both the mature and the extended precursor form of *TLC1* compared to *RRP4* cells (Fig. 5D). These data indicate that known RNA exosome target transcripts accumulate in *rrp4-G226D* cells and suggest that Rrp4 G226D impairs the function of the RNA exosome.

### The Rrp4 G226D variant causes broad transcriptomic changes

**FIGURE 6.**
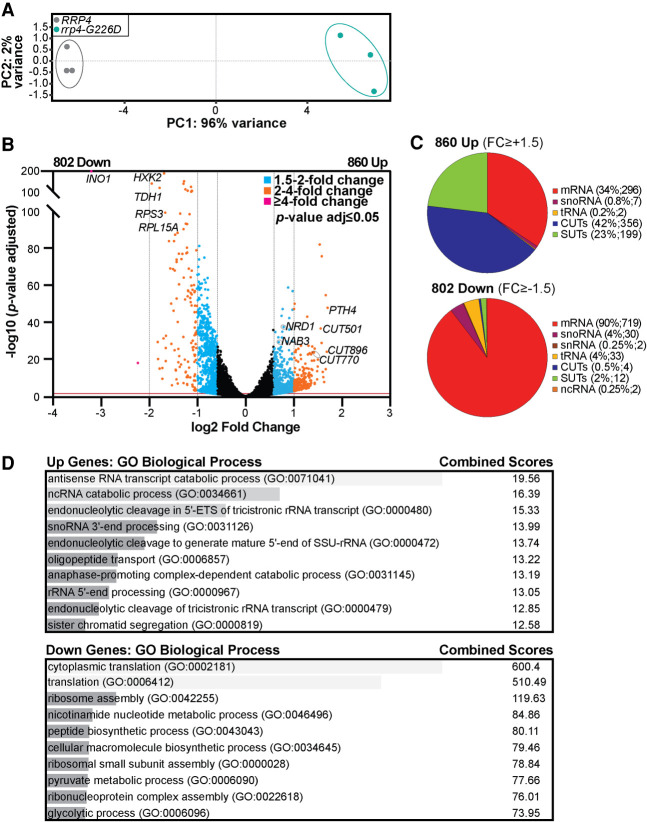
RNA-seq analysis of *rrp4-G226D* cells reveal distinct transcriptomic changes compared to *RRP4* cells. (*A*) Principal component analysis (PCA) of RNA-seq data collected from triplicate *RRP4* and *rrp4-G226D* cell samples shows that the gene expression patterns from independent *rrp4-G226D* samples are similar and thus cluster together, but are distinct from *RRP4* samples, which also cluster together. (*B*) A volcano plot of the differentially expressed transcripts in *rrp4-G226D* cells compared to *RRP4* cells shows that 860 transcripts are significantly Up and 802 transcripts are Down by 1.5-fold or more in *rrp4-G226D* cells. Statistically significant fold changes in transcript levels (Down or Up) in *rrp4-G226D* cells relative to *RRP4* cells are color coded (1.5–2 FC [blue]; 2–4 FC [orange]; ≥4 FC [purple]; *P*-value adjusted ≤0.05). Transcripts that were subsequently validated by RT-qPCR are labeled. (*C*) Pie charts of the percentages of different RNA classes within the 860 Up and 802 Down transcripts in *rrp4-G226D* cells reveal that increased transcripts are predominantly ncRNAs (CUTs; SUTs) and decreased transcripts are predominantly mRNAs. The RNA classes identified include messenger RNA (mRNA), small nuclear RNA (snRNA), small nucleolar RNA (snoRNA), transfer RNA (tRNA), cryptic unstable transcripts (CUTs; small, noncoding RNA), stable unannotated transcripts (SUTs; small, noncoding RNA), and other noncoding RNA (ncRNA; e.g., *TLC1*), (D) Gene ontology (GO) analysis for biological process in the Up and Down transcripts in *rrp4-G226D* cells reveals that ncRNA processing is significantly represented in the Up transcripts and translation is significantly represented in the Down transcripts. GO analysis was performed on coding (mRNA) and noncoding RNA (tRNAs, snoRNAs, and snRNAs) classes using the YeastEnrichr web server (Chen et al. 2013; Kuleshov et al. 2016, 2019). Gray bars represent the statistical significance of the biological process categories computed using combined score listed (log of the *P*-value from the Fisher exact test multiplied by the *z*-score of the deviation from the expected rank).

To further investigate the molecular consequences of the Rrp4 G226D substitution, we performed RNA-seq analysis on rRNA-depleted total RNA isolated from three independent biological replicates of the *rrp4-G226D* and control *RRP4* cells as described in Materials and Methods. Unbiased principal component analysis (PCA) of the resulting RNA-seq data produced two distinct clusters, indicating that the *rrp4* mutant transcriptome is distinct from the wild-type *RRP4* control (Fig. 6A). This separation between the two genotypes and reproducibility among the RNA-seq replicates allowed us to identify transcriptomic changes in *rrp4-G226D* mutant cells compared to the control (Fig. 6B). From differential gene expression analysis, we detect 860 transcripts increased (≥+1.5 Fold Change [FC], *P* < 0.05) and 802 transcripts decreased (≥−1.5 FC, *P* < 0.05) in *rrp4-G226D* cells compared to the *RRP4* control (Fig. 6B). Of the 860 transcripts increased, only a third are mRNAs (34%, 296 transcripts), with the majority being cryptic unstable transcripts (CUTs), stable unannotated transcripts (SUTs), and other ncRNAs (Fig. 6C). Consistent with the role the RNA exosome plays in degradation of nascent ncRNA species, the CUTs and SUTs combined make up the majority (65%) of transcripts that show a steady-state increase in *rrp4-G226D* cells (Fig. 6C). Of the 802 transcripts decreased, the majority are mRNAs (90%, 719 transcripts) (Fig. 6C), with the most significantly decreased transcript (≥−4 FC) being *INO1,* an mRNA that encodes inositol-3-phosphate synthetase (Donahue and Henry 1981; Klig and Henry 1984), which has previously been identified as a transcript bound to the catalytic subunit of the RNA exosome (Delan-Forino et al. 2017).

Gene Ontology (GO) analysis of the differentially expressed transcripts in *rrp4-G226D* cells using YeastEnrichr (Chen et al. 2013; Kuleshov et al. 2016, 2019) reveals that the ncRNA catabolic process is the most significant biological process category for the increased transcripts (Combined score 19.56) and cytoplasmic translation is the most significant category for the decreased transcripts (Combined score 600.4) (Fig. 6D). These GO analyses align with the transcripts that are altered, as two significantly decreased mRNAs (≥−1.5 FC), *RPS3* and *RPL15A,* encode components of the ribosome, and two significantly increased mRNAs (≥+1.5 FC), *NRD1* and *NAB3*, encode components of the Nrd1–Nab3–Sen1(NNS) transcription termination complex (Steinmetz and Brow 1998; Steinmetz et al. 2001; Wolin et al. 2012; Belair et al. 2018).

**FIGURE 7.**
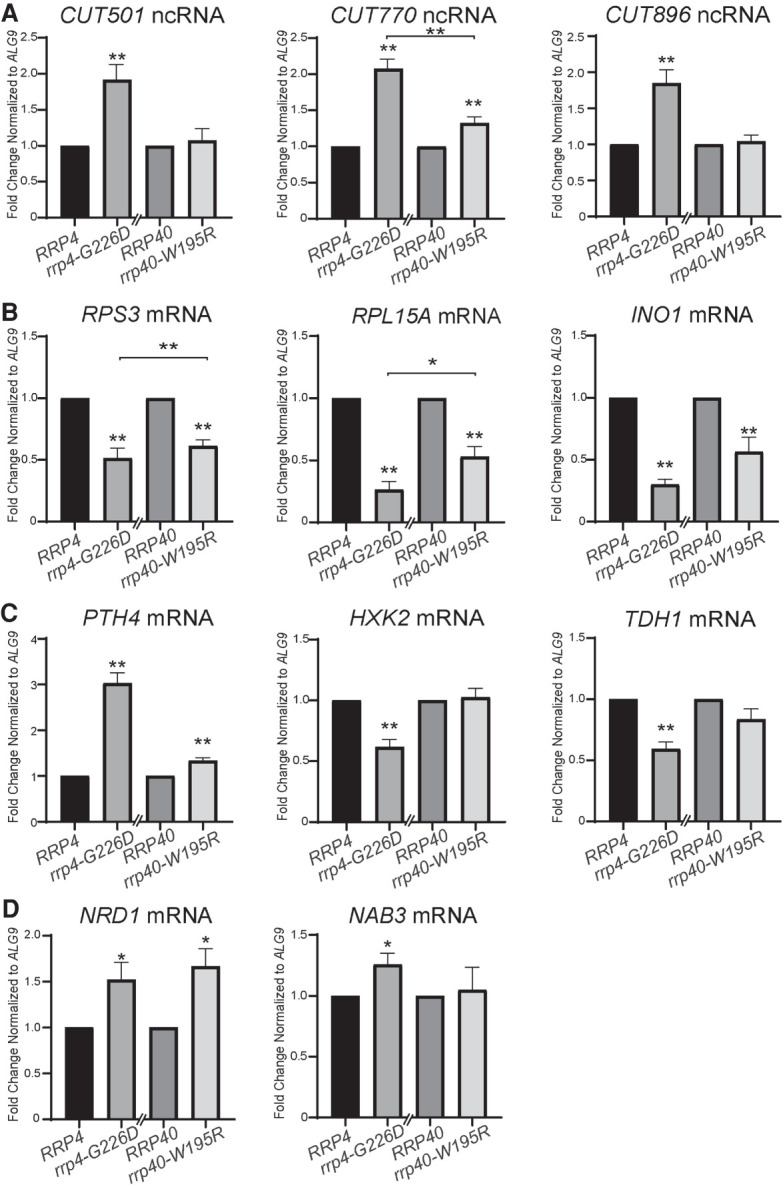
Validation of the differentially expressed transcripts identified in the RNA-seq confirms that the levels of key mRNAs and CUTs are significantly altered in *rrp4-G226D* cells and reveals that some of these transcripts are not changed in *rrp40-W195R* cells. (*A*) The steady-state levels of noncoding, cryptic unstable transcripts, *CUT501*, *CUT770*, and *CUT896*, are significantly increased in *rrp4-G226D* cells compared to control. The *CUT770* level is also increased but the *CUT501* and *CUT896* levels are not altered in *rrp40-W195R* cells. (*B*) The steady-state levels of ribosomal protein gene mRNAs, *RPS3* and *RPL15A*, and inositol-3-phosphate synthase mRNA, *INO1*, are significantly decreased in *rrp4-G226D* and *rrp40-W195R* cells relative to control *RRP4/40* cells. (*C*) The steady-state level of peptidyl tRNA hydrolase 4 mRNA, *PTH4*, is significantly increased in *rrp4-G226D* and *rrp40-W195R* cells relative to controls, whereas the levels of hexokinase isoenzyme 2 mRNA, *HXK2*, and glyceraldehyde-3-phosphate dehydrogenase isozyme 1 mRNA, *TDH1*, are significantly decreased in *rrp4-G226D* compared to control. The *HXK2* and *TDH1* levels are not altered in *rrp40-W195R* cells. (*D*) The steady-state levels of RNA exosome/termination cofactor mRNAs, *NRD1*, and *NAB3*, are significantly increased in *rrp4-G226D* cells compared to controls. The *NRD1* level is increased, but the *NAB3* level is not altered in *rrp40-W195R* cells. In *A*–*D*, total RNA was isolated from cells grown at 37°C and transcript levels were measured by RT-qPCR using gene specific primers (Supplemental Table S2), normalized relative to *RRP4/40,* and graphed as described in Materials and Methods. Error bars represent standard error of the mean from three biological replicates. Statistical significance of the RNA levels in *rrp4-G226D* and *rrp40-W195R* cells relative to control *RRP4/40* cells or between *rrp4/40* mutants is denoted by asterisks (*P-value ≤0.05; **P-value ≤0.01).

To validate altered gene expression detected in the RNA-seq analysis, we measured the levels of a subset of transcripts (Fig. 7). We performed this analysis on select coding and noncoding transcripts (labeled in Fig. 6B). This analysis confirms that the steady-state levels of three noncoding CUTs—*CUT501, CUT770, CUT896* (Fig. 7A)—and three mRNAs—*PTH4, NRD1, NAB3* (Fig. 7C,D)—that increased in the RNA-seq analysis are significantly increased (*P* < 0.05) in *rrp4-G226D* cells compared to *RRP4* control cells. We also validated decreased steady-state levels of several mRNAs (*RPS3*, *RPL15A*, *INO1*, *HXK2, TDH1)* (*P* < 0.01) in *rrp4-G226D* cells compared to control (Fig. 7B,C).

To compare the molecular consequences resulting from two pathogenic missense mutations in RNA exosome cap subunits, EXOSC2/Rrp4 and EXOSC3/Rrp40 (Fasken et al. 2017; Gillespie et al. 2017), we expanded the RT-qPCR analysis to include *rrp40-W195R* cells. Intriguingly, we found that some altered targets in *rrp4-G226D* cells are affected in both mutants, while others are significantly affected only in the *rrp4* mutant. The steady-state levels of *CUT501*, *CUT770*, and *CUT896* are only significantly increased in *rrp4-G226D* cells and not in *rrp40-W195R* cells (Fig. 7A). Steady-state levels of coding *RSP3*, *RPL15A*, and *INO1* mRNAs are significantly decreased in both *rrp-G226D* and *rrp40-W195R* cells compared to control cells (Fig. 7B). In contrast, the decrease in steady-state levels of *HXK2* and *TDH1* mRNAs is unique to the *rrp4-G226D* cells, as these coding RNAs are not affected in *rrp40-W195R* cells (Fig. 7C). The *PTH4* mRNA is significantly increased in *rrp40-W195R* cells compared to *RRP40* control, as observed in *rrp4-G226D* cells; however, the magnitude of the change detected was quite different. With respect to the NNS components, the *NRD1* steady-state level changes to a similar extent in both *rrp4-G226D* and *rrp40-W195R* cells compared to control; however, the significant increase in the *NAB3* steady-state level occurs only in *rrp4-G226D* cells (Fig. 7D).

### The *rrp4-G226D* mutant shows genetic interactions with nuclear RNA exosome cofactors

**FIGURE 8.**
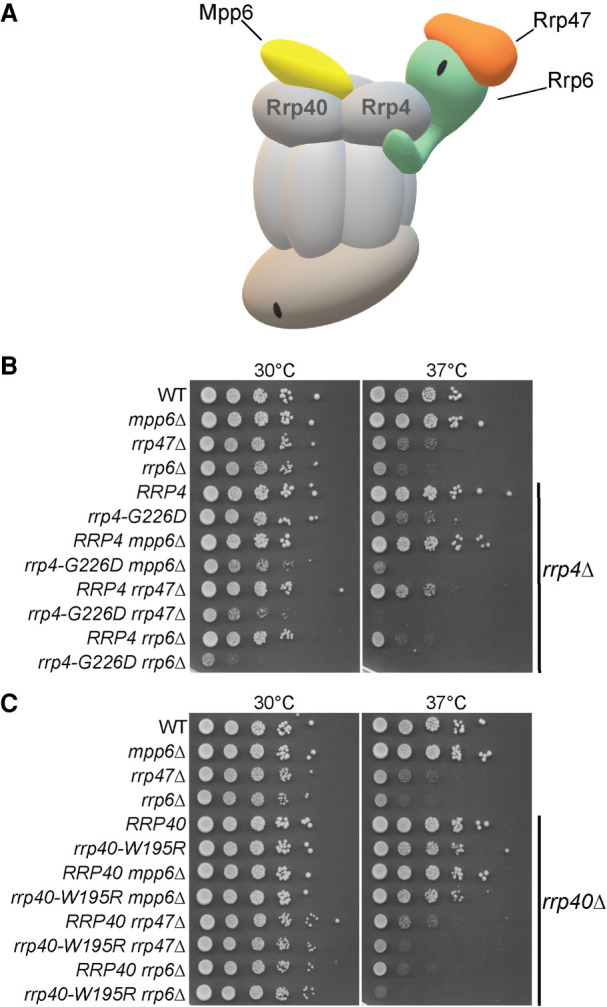
The *rrp4-G226D* mutant exhibits distinct negative genetic interactions with RNA exosome cofactor mutants that are not shared by the *rrp40-W195R* mutant. (*A*) Cartoon schematic of the budding yeast nuclear RNA exosome interacting with nuclear cofactors, Mpp6 and Rrp47, and the exoribonuclease Rrp6 (Schuller et al. 2018). (*B*) Double mutant cells containing *rrp4-G226D* and *mpp6*Δ, *rrp47*Δ, or *rrp6*Δ show impaired growth compared to single mutants at 30°C and 37°C. The double mutant cells (*rrp4*Δ with *mpp6*Δ, *rrp47*Δ, or *rrp6*Δ) containing control *RRP4* or *rrp4-G226D* plasmid were serially diluted, spotted onto solid media, and grown at the indicated temperatures for 3 d. (*C*) Double mutant cells containing *rrp40-W195R* and *mpp6*Δ do not exhibit a change in growth compared to single mutants, whereas double mutant cells containing *rrp40-W195R* and *rrp47*Δ or *rrp6*Δ show impaired growth compared to single mutants at 37°C. The double mutant cells (*rrp40*Δ with *mpp6Δ, rrp47*Δ, or *rrp6*Δ) containing control *RRP40* or *rrp40-W195R* plasmid were serially diluted, spotted onto solid media, and grown at indicated temperatures for 3 d.

The specificity of the RNA exosome for different RNA substrates is conferred by several interacting cofactors, which have been most extensively characterized in budding yeast (Schneider and Tollervey 2013; Zinder and Lima 2017). As depicted in Figure 8A, the exosome cofactor Rrp47 interacts with and stabilizes the exoribonuclease Rrp6, and the cofactor Mpp6 directly interacts with the nuclear RNA exosome (Schuch et al. 2014; Wasmuth et al. 2014, 2017). To determine whether the *rrp4-G226D* variant exhibits genetic interactions with RNA exosome cofactor mutants, we deleted the nonessential, nuclear exosome cofactor genes *MPP6*, *RRP47* and *RRP6* in combination with *rrp4-G226D*. For comparison, we also tested whether the *rrp40-W195R* variant shows genetic interactions with this series of mutants by deleting each gene in combination with *rrp40-W195R.* We examined the growth of these double mutants relative to single mutants (*rrp4-G226D* and *rrp40-*W195R) in solid media growth assays (Fig. 8). Interestingly, the *rrp4-G226D mpp6Δ, rrp4-G226D rrp6Δ,* and *rrp4-G226D rrp47*Δ double mutant cells all exhibit impaired growth compared to *rrp4-G226D* and single mutants at 30°C (Fig. 8B), indicating that deletion of *MPP6*, *RRP47*, or *RRP6* exacerbates the growth defect of *rrp4-G226D* cells. The impaired growth of the *rrp4-G226D rrp6*Δ double mutant is particularly striking. In contrast, *rrp40-W195R mpp6*Δ, *rrp40-W195R rrp47*Δ, and *rrp40-W195R rrp6*Δ double mutant cells do not show altered growth compared to *rrp40-W195R* or single mutant cells at 30°C (Fig. 8C).

The *rrp4-G226D* double mutants also exhibit enhanced growth defects relative to single mutants at 37°C. The impaired growth of the *rrp4-G226D mpp6*Δ double mutant at 37°C is particularly noteworthy as loss of *MPP6* does not alter cell growth at either 30°C or 37°C in single mutant cells or in double mutant *rrp40-W195R* cells (Fig. 8B,C). The *rrp40-W195R mpp6*Δ *rrp47*Δ and *rrp40-W195R rrp6*Δ double mutant cells exhibit impaired growth at 37°C, though not substantially worse when compared to the impaired growth of single mutants *rrp47*Δ or *rrp6*Δ at 37°C, as has been previously reported (Fig. 8B,C; Briggs et al. 1998; Mitchell et al. 2003).

### Rrp4 G226D has decreased association with the essential helicase Mtr4

**FIGURE 9.**
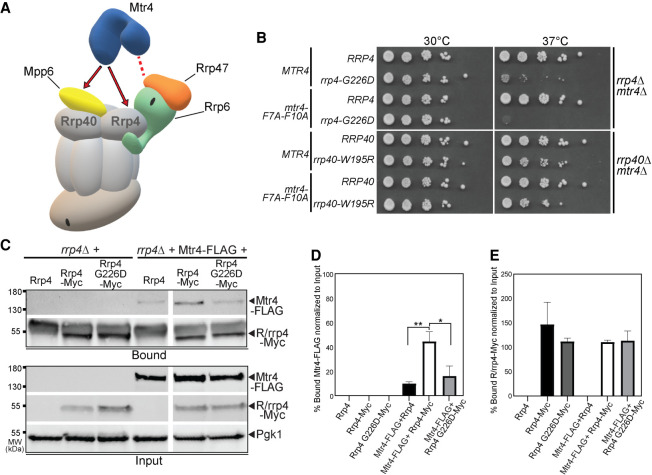
The *rrp4-G226D* mutant shows genetic interaction with an *mtr4* mutant that is impaired for interaction with Rrp6/Rrp47 and Rrp4 G226D impairs interaction with Mtr4. (*A*) Cartoon of the budding yeast nuclear RNA exosome depicting the molecular interactions that the essential RNA helicase, Mtr4, makes with the RNA exosome, and exosome cofactors (Schuch et al. 2014; Falk et al. 2017; Schuller et al. 2018). The association of the amino terminus of Mtr4 with the RNA exosome is facilitated by interactions with nuclear exosome cofactors, Rrp6/Rrp47 (denoted by the dashed red line). The association of Mtr4 with the RNA exosome is also facilitated by interactions with nuclear exosome cofactor, Mpp6, which is associated with the Rrp40 exosome subunit, and the Rrp4 exosome subunit (denoted by the solid red arrows). (*B*) Double mutant cells containing *rrp4-G226D* and *mtr4-F7A-F10A,* an *mtr4* mutant impaired for interaction with Rrp6/Rrp47, show lethality compared to the impaired growth of the single mutant *rrp4-G226D* at 37°C. In contrast, double mutant cells containing *rrp40-W195R* and *mtr4-F7A-F10A* show impaired growth at 37°C that is similar to the single mutant *rrp40-W195R*, which has been described previously (Fasken et al. 2017; Gillespie et al. 2017). The *rrp4Δ mtr4*Δ double mutant cells containing *RRP4* or *rrp4-G226D* plasmid and *rrp40Δ mtr4*Δ double mutant cells containing *RRP40* or *rrp40-W195R* plasmid that also harbor *MTR4* or *mtr4-F7A-F10A* plasmid were serially diluted, spotted onto solid media, and grown at the indicated temperatures for 3 d. (*C*) The Rrp4 G226D variant shows decreased association with Mtr4. Myc-tagged Rrp4 and Rrp4 G226D protein was immunoprecipitated from *rrp4*Δ cells coexpressing Rrp4-Myc and FLAG-tagged Mtr4 grown at 30°C using anti-Myc beads, and the amount of bound Mtr4-FLAG protein was detected by immunoblotting. The bound/input level of Mtr4-FLAG was detected with an anti-FLAG antibody, and the bound/input level of Rrp4-Myc was detected with an anti-Myc antibody. The input level of 3-phosphoglycerate kinase (Pgk1) was detected as a loading control. (*D*) Quantitation of the percentage of bound Mtr4-FLAG coimmunoprecipitated with Rrp4-Myc and Rrp4 G226D-Myc. Graph shows the mean percentage of bound Mtr4-FLAG from three independent experiments (*n* = 3). Error bars represent standard error of the mean. Statistical significance is denoted by asterisks (*P-value ≤0.05; **P-value ≤0.01). (*E*) Quantitation of percentage of bound Rrp4-Myc and Rrp4 G226D-Myc immunoprecipitated. Error bars represent standard error of the mean. The coimmunoprecipitations were performed and quantitated as described in Materials and Methods.

The nuclear exosome cofactors Mpp6 and Rrp47 and the associated exoribonuclease Rrp6 aid in recruiting the essential nuclear RNA helicase, Mtr4, to the RNA exosome (Fig. 9A; Wasmuth et al. 2017). Rrp6 and Rrp47 form a composite site that binds to the amino terminus of Mtr4, recruiting the helicase to the RNA exosome (Schuch et al. 2014). Mpp6 also tethers Mtr4 and Mtr4-containing complexes to the complex (Falk et al. 2017). Structural studies have also shown that human MTR4/MTREX and budding yeast Mtr4 directly interact with the RNA exosome by binding to a conserved region of EXOSC2/Rrp4 that also facilitates EXOSC10/Rrp6-RNA exosome interaction (Fig. 9A; Schuller et al. 2018; Weick et al. 2018). Given the negative genetic interactions observed between *rrp4-G226D* and nuclear cofactor mutants, and the binding interface between EXOSC2 and human MTR4 shown in structural studies (Weick et al. 2018), we tested whether the interaction between the RNA exosome and Mtr4 is affected in *rrp4-G226D* cells.

We tested for a genetic interaction between *rrp4-G226D* and *mtr4-F7A-F10A*, a mutant allele of *MTR4* that disrupts interactions with Rrp6/Rrp47 (Fig. 9B; Schuch et al. 2014). The *rrp4-G226D mtr4-F7A-F10A* double mutant cells grow similarly to *rrp4-G226D* cells at 30°C; however, the *rrp4-G226D mtr4-F7A-F10A* cells are not viable at 37°C (Fig. 9B). As a comparison, we performed a similar growth assay with *rrp40-W195R mtr4-F7A-F10A* double mutant cells and found that these cells show growth similar to *rrp40-W195R* cells at 30°C and 37°C (Fig. 9B). These data show that *rrp4-G226D* genetically interacts with a mutant allele of *MTR4* that disrupts interactions with Rrp6/Rrp47.

To investigate whether the physical interaction between Mtr4 and the RNA exosome is impacted by the Rrp4 G226D variant, we performed a coimmunoprecipitation with cells that express Rrp4-Myc or Rrp4 G226D-Myc as the sole copy of Rrp4 and coexpress Mtr4-FLAG (Fig. 9C–E). The Rrp4-Myc proteins were immunoprecipitated and association with Mtr4-FLAG was assayed by immunoblotting. Mtr4-FLAG coimmunoprecipitates with Rrp4-Myc but not with Rrp4 G226D-Myc (Fig. 9C). Results from three independent experiments are quantified for the amount of coisolated Mtr4-FLAG in Figure 9D. This difference in association of Mtr4-FLAG with wild-type Rrp4 versus Rrp4 G226D is not due to decreased protein levels or inefficient immunoprecipitation of Rrp4 G226D-Myc as quantitated in Figure 9E. Rather, these data demonstrate that Mtr4 association with the Rrp4 cap subunit is significantly disrupted by the Rrp4 G226D amino acid substitution (Fig. 9C,D). Combined with the genetic data (Fig. 9B), these results suggest that there is a disruption between Mtr4 and the RNA exosome complex in *rrp4-G226D* cells, thus providing a potential molecular mechanism for the impairment in RNA exosome caused by the Rrp4 G226D amino acid substitution.

## DISCUSSION

In this study, we modeled and analyzed pathogenic amino acid substitutions in the *S. cerevisiae* EXOSC2 ortholog, Rrp4. We generated *rrp4-G58V* and *rrp4-G226D* mutants, which correspond to the SHRF-linked mutations *EXOSC2-G30V* and *EXOSC2-G198D*, respectively. Analysis of the *rrp4-G58V* and *rrp4-G226D* cells reveals that these amino acid substitutions have distinct effects on RNA exosome function. The Rrp4-G58V variant is not able to function as the sole copy of the essential Rrp4 RNA exosome cap subunit as *rrp4-G58V* cells are not viable. In contrast, *rrp4-G226D* cells show a growth defect at 37°C. These *rrp4-G226D* cells show significant transcriptomic changes compared to control *RRP40* cells, including increases in steady-state levels of known direct RNA exosome targets such as precursors of 5.8S ribosomal RNA (rRNA), *U4* small nuclear RNA (snRNA), and *TLC1* telomerase RNA (Mitchell et al. 1996; Allmang et al. 1999; van Hoof et al. 2000; Davis and Ares 2006; Xu et al. 2009; Marquardt et al. 2011; Parker 2012; Schneider et al. 2012; Coy et al. 2013). RNA-seq analysis of *rrp4-G226D* cells show broad transcriptomic changes, with predominantly increased steady-state levels of noncoding RNA CUTs and SUTs that are usually regulated by nuclear surveillance mechanisms. The Rrp4 G226D variant can assemble into the RNA exosome, but both genetic and biochemical studies suggest interactions with key RNA exosome cofactors are impaired in *rrp4-G226D* cells. In particular, we observe decreased association of the essential helicase Mtr4 with Rrp4 G226D, suggesting a decreased interaction with this nuclear cofactor in vivo. Overall, these data suggest that the SHRF-linked pathogenic amino acid substitutions alter the overall function of the RNA exosome in vivo, resulting in defects in nuclear surveillance that may be due to impaired interaction with Mtr4. These results provide the first in vivo model of pathogenic amino acid substitutions that occur in EXOSC2.

In assessing the molecular and functional consequences of these *rrp4* variants in vivo, we first tested whether the modeled SHRF pathogenic amino acid substitutions affect protein levels and/or incorporation of the cap subunit into the RNA exosome. These data complement prior biochemical studies that used SHRF patient cells (Yang et al. 2019). Our biochemical assays suggest that Rrp4 G58V and Rrp4 G226D are not able to incorporate into the RNA exosome complex when a wild-type copy of the cap subunit is present (Fig. 4A); however, Rrp4 G226D incorporates into the complex when no competing wild-type subunit is present (Fig. 4B). These analyses suggest that the Rrp4 variants are outcompeted by a wild-type Rrp4 for incorporation into the complex, which is similar to previous studies of *rrp40-W195R* cells showing that Rrp40 variants cannot incorporate into the complex in the presence of a wild-type copy of Rrp40 and are subsequently targeted by the proteasome for degradation (Fasken et al. 2017). This reported decrease in protein half-life of unincorporated subunits into the complex could explain the decrease in steady-state level of Rrp4 G58V and Rrp4 G226D in cells expressing a wild-type *RRP4* copy (Fig. 3E). The lethality observed in *rrp4-G58V* cells when Rrp4 G58V is the sole copy of the essential cap subunit (Fig. 3A) could mean that Rrp4 G58V cannot associate with the RNA exosome, perhaps resulting in loss of functional complex *in vivo*. In contrast, *rrp4-G226D* cells are viable, but show temperature-sensitive growth.

Two of the three SHRF patients identified thus far are homozygous for the missense mutation *EXOSC2-G30V* (Di Donato et al. 2016), suggesting that this EXOSC2 variant is able to support RNA exosome function in humans. From our structural modeling, we predict similarities between both the EXOSC2–EXOSC4 and Rrp4–Rrp41 interface (Fig. 2A,C) and a conserved stabilizing salt bridge between the two RNA exosome subunits that depends on the Gly30 residue in EXOSC2 and Gly58 residue in Rrp4 (Supplemental Fig. S2). The EXOSC2–EXOSC4 and Rrp4–Rrp41 interfaces may be differentially impacted by the valine substitution in the two eukaryotic species which could account for the differences between budding yeast *rrp4-G58V* cells and homozygous *EXOSC2-G30V* patients. Previous studies also suggest that the RNA exosome plays an important role in tissue development and human embryonic stem cell differentiation (Belair et al. 2019; Yatsuka et al. 2020). The diverse clinical presentation in patients with SHRF could reflect these key developmental roles and/or different requirements in different cell types. Thus, the differential effects observed between budding yeast *rrp4-G58V* cells and *EXOSC2-G30V* patients could be indicative of differences in developmental time points or requirements between the two eukaryotes. Integrating additional disease models across other systems will be required to define how pathogenic missense mutations differentially impact RNA exosome function in a tissue-specific manner, leading to diverse disease pathologies.

RNA-seq analysis of the *rrp4-G226D* mutant cells revealed a broad spectrum of RNA classes that are altered in these mutant cells. The majority of the significantly increased transcripts in *rrp4-G226D* cells are comprised of the noncoding RNAs, CUTs, and SUTs (65% of all up transcripts with FC≥+1.5). As CUTs and SUTs are stabilized in RNA exosome mutants and cross-link to the RNA exosome (Wyers et al. 2005; Davis and Ares 2006; Gudipati et al. 2012; Schneider et al. 2012), we suggest that the elevated CUTs and SUTs observed in *rrp4-G226D* cells are due to impaired nuclear exosome function due to the Rrp4 G226D substitution. In contrast, the overwhelming majority of the significantly decreased transcripts are mRNAs (90% of all down transcripts with FC≥−1.5), with the most significantly decreased transcript being *INO1* mRNA. Previous studies have shown that *INO1* mRNA associates with the catalytic Dis3/Rrp44 subunit of the RNA exosome in budding yeast as determined by UV cross-linking and analysis of cDNA (CRAC) (Delan-Forino et al. 2017). This published data set also reports physical interaction of Dis3/Rrp44 with *HXK2, TDH1, RPL15A*, and *RPS3* mRNAs (Delan-Forino et al. 2017); other RNAs decreased in our RNA-seq analysis. The authors of this study suggested that these mRNA targets could be rapidly turned over in the cytoplasm by the RNA exosome, but our results show a decrease in these mRNAs, rather than the increase predicted if these transcripts were rapidly degraded by the RNA exosome. Notably, many ribosomal protein gene (*RPG*) mRNAs are decreased in *rrp4-G226D* cells and GO analysis of the decreased transcripts revealed cytoplasmic translation to be the most significantly affected biological process (Fig. 5D). Consistent with these data, decreases in *RPG* mRNAs have also been observed in *rrp6*Δ mutant cells, (Fox et al. 2015). Decreases of these specific mRNAs in *rrp4-G226D* cells could reflect dysregulation of the cytoplasmic RNA exosome in *rrp4-G226D* cells; however, it is not clear how these mRNA targets that physically associate with the catalytic subunit Rrp44 are decreased in *rrp4-G226D* cells. Overall, these data demonstrate that the Rrp4 G226D variant could alter both nuclear and cytoplasmic roles of the RNA exosome.

Many of the decreased mRNA transcripts observed in *rrp4-G226D* cells could result from indirect effects, reflecting cellular changes that occur when the function of the RNA exosome is compromised, leading to numerous downstream changes. Previous work in *Drosophila melanogaster* that used RNAi to deplete Rrp4 identified decreased levels of several transcripts encoding autophagy proteins (Yang et al. 2019). The authors postulated that defective autophagy could contribute to SHRF pathology (Yang et al. 2019). In our RNA-seq analysis of *rrp4-G226D* cells, we identified 16 autophagy transcripts that were decreased −1.5-fold (*P* < 0.05) (Supplemental Fig. S4), which is consistent with observations in this previous study. Further studies will be required to assess whether *rrp4-G226D* cells have impaired autophagy as well as to determine whether these mRNA transcripts are direct targets of regulation by the RNA exosome.

Both genetic and biochemical assays suggest that defects in the RNA exosome function in *rrp4-G226D* cells could be due to disrupted cofactor interactions, particularly decreased association with the essential RNA helicase Mtr4. Human structural studies have shown that MTR4/MTREX binds directly not only to MPH6/MPP6 but also to a conserved region of EXOSC2 (Weick et al. 2018). Perturbance of the MTR4/MTREX/Mtr4-RNA exosome interaction in humans and budding yeast could also impact association of the NEXT complex or TRAMP complex with the RNA exosome, thus affecting nuclear RNA quality control of several RNA classes, including processing of telomerase RNA, and degradation of cryptic ncRNAs (Houseley and Tollervey 2009). The increased levels of CUTs, SUTs*,* and precursors of *U4 snRNA* and *TLC1* observed in *rrp4-G226D* cells further lends support to suggest that the Mtr4-RNA exosome interaction is impaired by Rrp4 G226D. This finding is consistent with a previous study that used a structural and biochemical approach to study the budding yeast nuclear RNA exosome and the consequences of the pathogenic EXOSC3 W238R variant linked to PCH1b in the human RNA exosome (Falk et al. 2017). This study showed that substitution of Arg for Trp at position 238 (W238R) in EXOSC3, corresponding to Rrp40 W195R modeled in yeast, impaired the interaction with MPH6/MPP6. Taken together, our analysis of Rrp4 variants and the previous study of Rrp40 variants suggest that pathogenic amino acid substitutions in cap subunits could impair interactions with RNA exosome cofactors, suggesting a molecular mechanism that could underlie impaired RNA exosome function in vivo.

This study identified several in vivo consequences resulting from the Rrp4 G226D amino acid substitution. Biochemical, genetic, and RNA analyses show that *rrp4-G226D* cells have impaired RNA exosome function, broad transcriptomic changes and defects in RNA exosome cofactor interactions. When we compare these functional and molecular consequences in *rrp4-G226D* cells to *rrp40-W195R* cells we see some similarities and some differences. Both exosomopathy mutant models show growth defects, though *rrp4-G226D* cells show a more severe growth phenotype (Fig. 3B). In addition, both exosomopathy mutant models share changes in steady-state levels of some transcripts (Fig. 7B), but some transcripts show statistically significant changes only in *rrp4-G226D* cells. Genetic analyses also suggest that *rrp4-G226D* and *rrp40-W195R* mutants have similar negative genetic interactions with key nuclear exosome cofactor mutants (Fig. 8). However, we also detect a negative genetic interaction between *rrp4-G226D* and an *mtr4* mutant that is not observed with *rrp40-W195R*. Based on these observations, two possibilities present themselves. One possibility is that missense mutations in *RRP4* and *RRP40* have distinct functional consequences for RNA exosome activity, which would be consistent with the distinct clinical presentations in patients with these pathogenic variants. Alternatively, the *rrp4-G226D* allele may simply be a stronger allele than *rrp40-W195R*. A more extensive, comparison of additional exosomopathy mutant alleles will be required to distinguish between these two possibilities.

Utilizing the yeast genetic model system, we have begun to elucidate the functional consequences that result from the pathogenic amino acid substitutions in EXOSC2 in SHRF patients. By modeling these mutations in the corresponding *RRP4* gene*,* we have generated a system that can be used to understand how pathogenic amino acid substitutions impact the function of the RNA exosome. This study also adds to the growing collection of in vivo RNA exosomopathy mutant models that can be compared to one another to define the in vivo consequences resulting from each mutation. For several RNA exosomopathies, including SHRF syndrome, the patient population is quite small, making analysis of patient tissue samples challenging if not impossible. Our findings presented here can be integrated into the body of work describing the SHRF *EXOSC2* mutations, further expanding our understanding of the unique disease pathology. Future comparative in vivo analysis of exosomopathy models will allow for deeper understanding of how diverse clinical symptoms are linked to changes in a single molecular machine. Furthermore, this type of in vivo comparison may shed light on the basic biology of the RNA exosome, as many questions still exist regarding RNA substrate targeting and regulation of this essential molecular machine. This study not only provides the first in vivo study that models *EXOSC2* mutations identified in SHRF patients, but also provides a platform for the first direct comparison of the consequences of pathogenic missense mutations in genes encoding cap subunits of the RNA exosome.

## MATERIALS AND METHODS

### Chemicals and media

All chemicals were obtained from Sigma-Aldrich, United States Biological, or Fisher Scientific unless otherwise noted. All media were prepared by standard procedures (Adams et al. 1997).

### Protein structure analysis

We used the cryo-EM structure (PDB 6D6R) of the human nuclear RNA exosome at 3.45 Å resolution (Weick et al. 2018) and the cryo-EM structure (PDB 6FSZ) of the budding yeast nuclear RNA exosome at 4.6 Å (Schuller et al. 2018). Structural modeling was performed using the PyMOL viewer (The PyMOL Molecular Graphics System, Version 2.0 Schrödinger, LLC). The mCSM-PP12 (Rodrigues et al. 2019), Polymorphism Phenotyping v2 (PolyPhen-2) (Adzhubei et al. 2010), Protein Variation Effect Analyzer (PROVEAN) (Choi 2012; Choi et al. 2012), and SNAP-2 (Hecht et al. 2015) webservers were used for predicting the effects of the *EXOSC2* mutations on protein stability and function.

### *Saccharomyces cerevisiae* strains and plasmids

All DNA manipulations were performed according to standard procedures (Sambrook et al. 1989). *S. cerevisiae* strains and plasmids used in this study are listed in Supplemental Table S1. The *rrp4*Δ (yAV1103) and *rrp40*Δ (yAV1107) strains were previously described (Schaeffer et al. 2009; Losh 2018). The *RRP43-TAP* (ACY2788) strain was obtained from Horizon Discovery Biosciences Limited and was previously described (Ghaemmaghami et al. 2003). The *RRP43-TAP rrp4*Δ (ACY2803) strain was constructed by deletion of the genomic *RRP4* ORF in the *RRP43-TAP* (ACY2788) strain containing a *RRP4 URA3* maintenance plasmid by homologous recombination using a *RRP4-UTR neoMX4* PCR product. The *rrp4Δ mpp6*Δ (ACY2471), *rrp4Δ rrp47*Δ (ACY2474), *rrp4Δ rrp6*Δ (ACY2478) strains and the *rrp40Δ mpp6*Δ (ACY2638), *rrp40Δ rrp47*Δ (ACY2462), *rrp40Δ rrp6*Δ (ACY2466) strains were constructed by deletion of the *MPP6*, *RRP47*, and *RRP6* ORF in the *rrp4*Δ (yAV1103) and *rrp40*Δ (yAV1107) strains by homologous recombination using *MPP6*-, *RRP47*-, or *RRP6-UTR natMX4* PCR products. The *rrp4Δ mtr4*Δ (ACY2536) and *rrp40Δ mtr4*Δ (ACY2540) strains were constructed by consecutive deletion of the genomic *MTR4* ORF and *RRP4* ORF or *RRP40* ORF in a wild-type (W303) strain harboring a [-*MTR4; RRP4; URA3*] (pAC3714) or [*MTR4; RRP40; URA3*] (pAC3713) maintenance plasmid by homologous recombination using *MTR4-UTR natMX4* and *RRP4-UTR* or *RRP40-UTRneoMX4* PCR products. Construction of *RRP40-2*×*Myc* and *rrp40-2*×*Myc* variant plasmids (pAC3161, pAC3162, and pAC3259) was reported previously (Fasken et al. 2017). The *RRP4-2*×*Myc LEU2 CEN6* (pAC3474) plasmid was constructed by PCR amplification of the endogenous promoter, 5′-UTR and ORF of the *RRP4* gene from *S. cerevisiae* genomic DNA and cloning into pRS315 plasmid containing a carboxy-terminal 2×Myc tag and the *ADH1* 3′-UTR (Sikorski and Hieter 1989). The *rrp4-G58V-2*×*Myc* (pAC3476) and *rrp4-G226D-2*×*Myc* (pAC3477) plasmids were generated by site-directed mutagenesis of the *RRP4-2*×*Myc* (pAC3474) plasmid using oligonucleotides containing the SHRF syndrome-linked G58V and G226D missense mutations and the QuikChange II Site-Directed Mutagenesis Kit (Agilent). The untagged *RRP4/rrp4-G226D* (pAC3656, pAC3659) and *RRP40/rrp40-W195R* (pAC3652, pAC3655) plasmids and Myc-tagged *RRP4/rrp4-G226D* (pAC3669, pAC3672) plasmid containing native 3′-UTRs were generated by excision of the *2*×*Myc*-*ADH1* 3′-UTR from each *RRP4/40-Myc LEU2 CEN6* plasmid by restriction digestion and cloning of the native *RRP4* 3′-UTR, *RRP40* 3′-UTR, or *2*×*Myc-RRP4 3*′*-UTR* PCR product into the relevant plasmid using NEBuilder HiFi Assembly (New England BioLabs). The -*MTR4 HIS3 CEN6* (pAC4096) plasmid was constructed by PCR amplification of the endogenous promoter, 5′-UTR, ORF, and 3′UTR of the *MTR4* gene from *S. cerevisiae* genomic DNA and cloning into pRS313 (Sikorski and Hieter 1989). The *mtr4-F7A-F10A* (pAC4099) plasmid was generated by site-directed mutagenesis of the *MTR4 HIS CEN6* plasmid (pAC4096) using oligonucleotides containing the F7A and F10A missense mutations and the QuikChange II Site-Directed Mutagenesis Kit (Agilent). The *MTR4-2*×*FLAG* (pAC3719) plasmid was constructed by PCR amplification of the *MTR4* promoter-5′-UTR-ORF (No Stop) and *2*×*FLAG-native MTR4* 3′-UTR using *MTR4* template plasmid (pAC2897 [Fasken et al. 2011]) and oligonucleotides encoding 2×FLAG epitopes and cloning into the pRS313 plasmid (Sikorski and Hieter 1989).

### *Saccharomyces cerevisiae* transformations and growth assays

All yeast transformations were performed according to the standard Lithium Acetate (LiOAc) protocol (Burke et al. 2000). Cells were grown overnight to saturation in a 30°C incubator in liquid YEPD (1% yeast extract, 2% peptone, 2% dextrose, in distilled water). Cell concentrations were normalized to an OD_600_ = 0.4 in 10 mL YEPD and incubated at 30°C for 5 h. The cells were washed with TE/LiOAc and resuspended in TE/LiOAc to a concentration of 2 × 10^9^ cells/mL. To the cells (50 µL), plasmid DNA, single-stranded carrier DNA (5 µL), and PEG/TE/LiOAc (300 µL) were added and cells were agitated for 30 mins at 30°C. DMSO (35 µL) was added and the cells were heat shocked at 42°C for 15 min. The cells were washed and plated onto selective media.

To test the in vivo function of the *rrp4* variants that model the *EXOSC2* variants in SHRF syndrome, a standard plasmid shuffle assay was used. The *rrp4*Δ (yAV1103) cells containing a *RRP4 URA3* maintenance plasmid and transformed with vector (pRS315), *RRP4-2*×*Myc* (pAC3474), *rrp4-G8A-2*×*Myc* (pAC3476), or *rrp4-G226D-2*×*Myc* (pAC3477) plasmid were grown overnight and serially diluted and spotted onto Ura^−^ Leu^−^ minimal media plates, which select for cells that contain both the *RRP4 URA3* maintenance plasmid and the *RRP4*/*rrp4 LEU2* plasmid, and 5-FOA Leu^−^ minimal media plates, which select for cells that lack the *RRP4 URA3* maintenance plasmid and contain only the *RRP4*/*rrp4 LEU2* plasmid. The plates were incubated at 30°C and 37°C for 2 d.

The in vivo function of the *rrp4-G226D* variant and the *rrp40-W195R* variant was assessed in growth assays on solid media and in liquid culture. For growth on solid media, *rrp4*Δ (yAV1103) cells containing only *RRP4* (pAC3656) or *rrp4-G226D* (pAC3659) and *rrp40*Δ (yAV1107) cells containing only *RRP40* (pAC3652) or *rrp40-W195R* (pAC3655) were grown in 2 mL Leu^−^ minimal media overnight at 30°C to saturation. Cell concentrations were normalized to an OD_600_ = 0.5, serially diluted in 10-fold dilutions, spotted onto Leu^−^ minimal media plates, and grown at 25°C, 30°C, and 37°C for 2–3 d. For growth in liquid culture, cells were grown in 2 mL Leu^−^ minimal media overnight at 30°C to saturation, diluted to an OD_600_ = 0.01 in Leu^−^ minimal media in a 24-well plate, and growth at 37°C was monitored and recorded at OD_600_ in a BioTek Synergy Mx microplate reader with Gen5 v2.04 software over 24 h. Technical triplicates of each strain were measured, and the average of these triplicates was calculated and graphed.

### Immunoblotting

For analysis of carboxy-terminally Myc-tagged Rrp4 protein expression levels, *rrp4*Δ (yAV1103) cells expressing only Rrp4-2×Myc (pAC3669) or rrp4-G226D-2×Myc (pAC3672) were grown in 2 mL Leu^−^ minimal media overnight at 30°C to saturation, and 10 mL cultures with an OD_600_ = 0.2 were prepared and grown at 30°C and 37°C for 5 h. Additionally, *rrp4*Δ (yAV1103) cells containing *RRP4 URA3* maintenance plasmid and expressing Rrp4-2×Myc (pAC3474), rrp4-G58V (pAC3476), or rrp4-G226D-2×Myc (pAC3477) were grown in 2 mL Ura^−^ Leu^−^ minimal media overnight at 30°C, and 10 mL cultures with an OD_600_ = 0.2 were prepared and grown at 30°C for 5 h. Cell pellets were collected by centrifugation, transferred to 2 mL screw-cap tubes and stored at −80°C. Yeast cell lysates were prepared by resuspending cell pellets in 0.3–0.5 mL of RIPA-2 Buffer (50 mM Tris-HCl, pH 8; 150 mM NaCl; 0.5% sodium deoxycholate; 1% NP40; 0.1% SDS) supplemented with protease inhibitors (1 mM PMSF; Pierce Protease Inhibitors [Thermo Fisher Scientific]), and 300 µL of glass beads. Cells were disrupted in a Mini-Beadbeater 16 Cell Disrupter (BioSpec) for 4 × 1 min at 25°C with 1 min on ice between repetitions, and then centrifuged at 16,000*g* for 15 min at 4°C. Protein lysate concentration was determined by Pierce BCA Protein Assay Kit (Life Technologies). Whole cell lysate protein samples (40 µg) in reducing sample buffer (50 mM Tris HCl, pH 6.8; 100 mM DTT; 2% SDS; 0.1% Bromophenol Blue; 10% Glycerol) were resolved on 4%–20% Criterion TGX Stain-Free Precast Polyacrylamide Gels (Bio-Rad), transferred to nitrocellulose membranes (Bio-Rad), and Myc-tagged Rrp4 proteins were detected with anti-Myc monoclonal antibody 9B11 (1:2000; Cell Signaling). The 3-phosphoglycerate kinase (Pgk1) protein was detected using anti-Pgk1 monoclonal antibody (1:30,000; Invitrogen) as a loading control.

### Quantitation of immunoblotting

The protein band intensities/areas from immunoblots were quantitated using ImageJ v1.4 software (National Institutes of Health, MD; http://rsb.info.nih.gov/ij/) and mean fold changes in protein levels were calculated in Microsoft Excel (Microsoft Corporation). To quantitate the mean fold change in Rrp4 G226D-Myc variant level relative to wild-type Rrp4-Myc level in *rrp4*Δ cells grown at 30°C and 37°C from three immunoblots (Fig. 3D) or the fold change in rrp4-G58V-Myc and rrp4-G226D-Myc level in *rrp4*Δ cells containing untagged *RRP4* from three immunoblots (Fig. 3E), R/rrp4-Myc intensity was first normalized to loading control Pgk1 intensity and then normalized to wild-type Rrp4-Myc intensity at 30°C or 37°C for each immunoblot. The mean fold change in R/rrp4-Myc level relative to Rrp4-Myc and standard error of the mean are graphed in Figure 3F and G. To quantitate the mean percent bound of Mtr4-FLAG coimmunoprecipitated with Rrp4-Myc or Rrp4 G226D-Myc (Fig. 9C), background intensity of Mtr4-FLAG was subtracted from the bound Mtr4-FLAG intensity. Then bound and input Mtr4-FLAG intensity was normalized to input Pgk1 intensity. Percent bound was calculated by dividing normalized bound Mtr4-FLAG by normalized input Mtr4-FLAG. To quantitate the mean percent bound Rrp4-Myc or Rrp4 G226D-Myc that immunoprecipitates (Fig. 9C), bound and input R/rrp4-Myc was normalized to input Pgk1 intensity. Percent bound was calculated by dividing normalized bound R/rrp4-Myc intensity by normalized input R/rrp4-Myc intensity. Mean percent bound Mtr4-FLAG and R/rrp4-Myc and standard error of the mean are graphed in Figure 9D and E.

### Northern blotting

For analysis of 5.8S pre-rRNA processing—detection of 7S pre-rRNA and processing intermediates—in *rrp4* and *rrp40* mutant cells, *rrp4*Δ (yAV1103) cells containing *RRP4-2*×*Myc* (pAC3474) or *rrp4-G226D-2*×*Myc* (pAC3477) and *rrp40*Δ (yAV1107) cells containing *RRP40-2*×*Myc* (pAC3161), *rrp40-G8A-2*×*Myc* (pAC3162), or *rrp40-W195R-2*×*Myc* (pAC3259) were grown in 2 mL Leu^−^ minimal media overnight at 30°C, 10 mL cultures with an OD_600_ = 0.4 were prepared and grown at 37°C for 5 h. Cells were collected by centrifugation (2163*g*), transferred to 2 mL screw cap tubes and stored at −80°C. Total RNA from cells was resolved on a Criterion TBE-Urea Polyacrylamide Gel (Bio-Rad), blotted to a nylon membrane and membrane was probed with radiolabeled 5.8S-ITS2 rRNA (boundary) oligonucleotide (AC4211/Probe 020-5′-TGAGAAGGAAATGACGCT) to detect 7S pre-rRNA and intermediates and stained with methylene blue stain to visualize 5.8S rRNA as a loading control. Total RNA (5 µg) was mixed with equal volume of RNA loading dye (1×TBE; 12% Ficoll; 7M Urea; 0.01 bromophenol blue; 0.02% xylene cyanol) and resolved on 10% TBE-Urea Polyacrylamide Gel in 1×TBE at 200V for 1.5 h. RNA was transferred to Hybond-N+ nylon membrane (Amersham, GE Healthcare) at 15 V for 100 min in 1×TBE and cross-linked to membrane with UV light (120,000 µJoules) using UV Stratalinker 2400 (Stratagene). The membrane was incubated in Rapid-hyb hybridization buffer (Amersham, GE healthcare) at 37°C for 1 h. DNA oligonucleotide (100 ng) was 5′-end labeled with [γ-P32]-ATP (PerkinElmer) using polynucleotide kinase (New England Biolabs) at 37°C for 30 min. [P32]-Labeled oligonucleotide probe was purified through a G25 microspin column (GE Healthcare), heated at 100°C for 5 min, and added to hybridization buffer. The oligonucleotide probe was hybridized to membrane in hybridization buffer at 37°C overnight. Following removal of hybridization buffer, membrane was rinsed twice in 5× SSPE; 0.1% SDS at 25°C and washed twice in 0.5× SSPE; 0.1% SDS at 37°C for 20 min each. The membrane was exposed to phosphoscreen overnight and imaged using Typhoon FLA 7000 phosphorimager (GE Healthcare).

### Total RNA isolation

Total RNA from *S. cerevisiae rrp4* and *rrp40* mutant cells was isolated using TRIzol (Invitrogen) for qRT-PCR and northern blotting and MasterPure Yeast RNA Purification Kit (Epicentre, Lucigen) for RNA-seq. *S. cerevisiae* cells were grown in 2 mL Leu^−^ minimal media overnight at 30°C to saturation. Cultures were diluted in 10 mL Leu^−^ minimal media to an OD_600_ = 0.2 and grown for 5 h at 37°C. Cells were pelleted by centrifugation, transferred into 2 mL screw cap tubes, and stored at −80°C. To prepare total RNA using TRIzol, cells were resuspended in 1 mL TRIzol (Invitrogen) with 300 µL of glass beads. Cell samples were disrupted in a BioSpec Mini-Beadbeater 16 Cell Disrupter for 2 min at 25°C. For each sample, 100 µL of 1-bromo-3-chloropropane (BCP) was added, sample was vortexed for 15 sec, and incubated at 25°C for 2 min. The sample was centrifuged at 16,300*g* for 8 min at 4°C, and the upper layer was transferred to a fresh microfuge tube. RNA was precipitated with 500 µL isopropanol and sample was vortexed for 10 sec to mix. Total RNA was pelleted by centrifugation at 16,300*g* for 8 min at 4°C. The RNA pellet was washed with 1 mL 75% ethanol, centrifuged at 16,300*g* for 5 min at 4°C, and air-dried for 15 min. Total RNA was resuspended in 50 µL diethylpyrocarbonate (DEPC, Sigma)-treated water and stored at −80°C. Total RNA was prepared using MasterPure Yeast RNA Purification Kit (Epicentre, Lucigen) according to manufacturer's protocol. Total RNA was resuspended in 50 µL DEPC-treated water and stored at −80°C.

### qRT-PCR

All oligonucleotides used in this study are summarized in Supplemental Table S2. For analysis of steady-state RNA levels using quantitative PCR, three independent biological replicates of *rrp4*Δ (yAV1103) cells containing only *RRP4* (pAC3656) or *rrp4-G226D* (pAC3659) and *rrp40*Δ (yAV1107) cells containing only *RRP40* (pAC3652) or *rrp40-W195R* (pAC3655) were grown in 2 mL Leu^−^ minimal media overnight at 30°C, 10 mL cultures with an OD_600_ = 0.2 were prepared and cells were grown at 37°C for 5 h. Total RNA was isolated from cell pellets and 1 µg of total RNA was reverse transcribed to first strand cDNA using the M-MLV Reverse Transcriptase (Invitrogen) according to manufacturer's protocol. Quantitative PCR was performed on technical triplicates of cDNA (10 ng) from three independent biological replicates using gene specific primers (0.5 mM; Supplemental Table S2), QuantiTect SYBR Green PCR master mix (Qiagen) on a StepOnePlus Real-Time PCR machine (Applied Biosystems; Tanneal = 55°C, 44 cycles). *ALG9* was used as an internal control. The mean RNA levels were calculated by the ΔΔCt method (Livak and Schmittgen 2001). Mean levels of RNA calculated in mutant cells are normalized to mean levels in wild-type cells and converted and graphed as an RNA fold change relative to wild-type. All primers used are summarized in Supplemental Table S2.

### RNA-seq analysis

RNA-seq was performed on three independent biological replicates of *rrp4*Δ (yAV1103) cells containing *RRP4-2*×*Myc* (pAC3474) or *rrp4-G226D-2*×*Myc* (pAC3477) as the sole copy of *RRP4* grown at 37°C. Cells were grown in 2 mL Leu^−^ minimal media overnight at 30°C, diluted to an OD_600_ = 0.4 in 10 mL Leu^−^ minimal media, grown at 37°C for 5 h, and collected and stored at −80°C. Total RNA was isolated, rRNA was depleted, and stranded cDNA libraries were prepared using TruSeq Total RNA Stranded Library Prep kit (Illumina). Paired-end sequencing of the cDNA libraries was performed on a HiSeq4000 instrument (2 × 150 cycles) at Frederick National Laboratory for Cancer Research (FNLCR) at the CCR Sequencing Facility, NCI, NIH. The *RRP4* samples yielded an average of 28,890,739 pass filter reads and the *rrp4-G226D* samples yielded an average of 34,644,683 pass filter reads, with a base call quality of 94% of bases with Q30 and above. The reads were mapped to the *S. cerevisiae* S288C genome assembly R64-1-1, annotated with CUTs and SUTs (Xu et al. 2009), using the STAR RNA-seq aligner (v2.7.5b [Dobin et al. 2012]). The reads per gene feature were counted using featureCounts (v1.6.4+galaxy2 [Liao et al. 2014]). Differential gene expression analysis on raw read counts was performed using DESeq2 (v2.11.40.6+galaxy1 [Love et al. 2014]) to identify genes significantly changed (*P*-value <0.05, ≥1.5 fold change) in *rrp4-G226D* samples relative to *RRP4* samples. Principal component analysis (PCA) on raw read counts was also performed using DESeq2. A volcano plot of differential gene expression data was produced in Prism 8 (Graphpad Software). Piecharts of RNA class percentages in significantly altered genes were generated in Microsoft Excel for Mac (Microsoft Corp.). Gene Ontology (GO) analysis on significantly altered genes for Biological Process category was performed using the YeastEnrichr webserver (http://amp.pharm.mssm.edu/YeastEnrichr/ [Kuleshov et al. 2019]).

### Genetic interaction analysis

To test genetic interactions between *rrp4-G226D* or *rrp40-W195R* and RNA exosome cofactor/subunit deletion mutants, *rrp4Δ mpp6Δ* (ACY2471), *rrp4Δ rrp47Δ* (ACY2474), and *rrp4Δ rrp6Δ* (ACY2478) cells containing only *RRP4* (pAC3656) or *rrp4-G226D* (pAC3659) and *rrp40Δ mpp6Δ* (ACY2638), *rrp40Δ rrp47Δ* (ACY2462), and *rrp40Δ rrp6Δ* (ACY2466) cells containing only *RRP40* (ACY3652) or *rrp40-W195R* (ACY3655) were grown in 2 mL Leu^−^ minimal media overnight at 30°C to saturation, serially diluted, and spotted on Leu^−^ minimal media plates. The plates were incubated at 30°C and 37°C for 3 d.

To test genetic interactions between *rrp4-G226D* or *rrp40-W195R* and the *mtr4* mutant, *mtr4-F7A-F10A*, *rrp4Δ mtr4Δ* (ACY2536) cells containing the [*MTR4; RRP4; URA3*] (pAC3714) maintenance plasmid were transformed with *RRP4* (pAC3656) or *rrp4-G226D* (pAC3659) *LEU2* plasmid, and *rrp40Δ mtr4Δ* (ACY2540) cells containing the [*MTR4; RRP40; URA3*] (pAC3713) maintenance plasmid were transformed with *RRP40* (pAC3652) or *rrp40-W195R* (pAC3655) *LEU2* plasmid and selected on Ura^−^Leu^−^ minimal media plates. Transformed cells containing both the *URA3* maintenance plasmid and the *RRP4*/*rrp4-G226D* or *RRP40*/*rrp40-W195R LEU2* plasmid were subsequently transformed with *MTR4* (pAC4096) or *mtr4-F7A-F10A* (pAC4099) *HIS3* plasmids and selected on Ura^−^Leu^−^His^−^ minimal media plates. The transformed cells were then streaked onto 5-FOA Leu^−^ His^−^ plates to select for cells that did not contain the *URA3* maintenance plasmid. The resulting *rrp4Δ mtr4Δ* cells containing only *RRP4* or *rrp4-G226D LEU2* plasmid and *MTR4* or *mtr4-F7A-F10A HIS3* plasmid and *rrp40Δ mtr4Δ* cells containing only *RRP40* or *rrp40-W195R LEU2* plasmid and *MTR4* or *mtr4-F7A-F10A HIS3* plasmid were grown in 2 mL Leu^−^ His^−^ minimal media overnight at 30°C to saturation, serially diluted, and spotted on Leu^−^ His^−^ minimal media plates. The plates were incubated at 30°C and 37°C for 3 d.

### Coimmunoprecipitations

All immunoprecipitations were performed using the same protocol. Cell samples were grown in 2 mL selective media overnight at 30°C to saturation and 10–20 mL cultures with an OD_600_ = 0.2–0.3 were prepared and grown at 30°C for 5 h. Yeast cell lysates were prepared by resuspending cell pellets in 0.5–0.75 mL of IPP150 Buffer (10 mM Tris-HCl, pH 8; 150 mM NaCl; 0.1% NP40, PMSF) supplemented with protease inhibitors (1 mM PMSF; Pierce Protease Inhibitors [Thermo Fisher Scientific]), and 300 µL of glass beads. Cells were disrupted in a Mini-Beadbeater 16 Cell Disrupter (BioSpec) for 4–5 × 1 min at 25°C with 1 min on ice between repetitions. Crude lysate was transferred to a chilled microcentrifuge tube and remaining beads were washed with an additional 150 µL of IPP150 Buffer. Lysate was then cleared by centrifugation at 16,000*g* for 10 min at 4°C. Protein lysate concentration was determined by Pierce BCA Protein Assay Kit (Life Technologies). For input samples, 40 µg of cleared lysate was collected and frozen at −20°C. For coimmunoprecipitations, 1 mg of cleared lysate in 1 mL IPP150 Buffer was prepared, 15–30 µL of a 1:1 bead slurry of either Pierce Anti-c-Myc Magnetic Beads (Thermo Fisher) or IgG Sepharose 6 Fast Flow Beads (GE Healthcare) was added, and samples were incubated at 4°C overnight with mixing. After overnight incubation, beads were washed three times in 1 mL IPP150 Buffer for 15 sec each (anti-Myc beads) or 5 min each (IgG Sepharose beads). Whole cell lysate input samples (40 µg) and total bound samples in reducing sample buffer were boiled for 5 min at 100°C, resolved on 4%–20% Criterion TGX Stain-Free Precast Polyacrylamide Gels (Bio-Rad), transferred to nitrocellulose membranes (Bio-Rad) and immunoblotted. Myc-tagged Rrp4 proteins were detected with mouse anti-Myc monoclonal antibody 9B11 (1:2000; Cell Signaling). TAP-tagged Rrp43 protein was detected with peroxidase anti-peroxidase (PAP) soluble complex antibody produced in rabbit (1:5000, Sigma-Aldrich). FLAG-tagged Mtr4 protein was detected with anti-DYKDDDDK (FLAG) tag rabbit monoclonal antibody D6W5B (1:2000; Cell Signaling). The 3-phosphoglycerate kinase (Pgk1) protein was detected using anti-Pgk1 monoclonal antibody (1:30,000; Invitrogen) as a loading control.

To assess association of Rrp4 G226D with the RNA exosome complex, we utilized *RRP43-TAP* (YCR035C) cells expressing *RRP4-Myc* (pAC3669)*, rrp4-G58V-Myc* (pAC3670) or *rrp4-G226D-Myc* (pAC3672) and *RRP43-TAP rrp4Δ* (ACY2803) cells expressing only *RRP4-Myc* (pAC3669) or *rrp4-G226D-Myc* (pAC3672) and immunoprecipitated Rrp43-TAP using the IgG Sepharose beads. Levels of associated Rrp4-Myc proteins with the Rrp43-TAP tagged subunit were detected by immunoblotting. To assess association of Rrp4 G226D with the Mtr4 helicase, we utilized *rrp4Δ* (yAV1103) cells expressing only *RRP4-Myc* (pAC3669) or *rrp4-G226D-Myc* (pAC3672) and coexpressing exogenous *MTR4-FLAG* (pAC3719) and immunoprecipitated Rrp4-Myc or Rrp4 G226D-Myc using anti-c-Myc beads. Levels of associated Mtr4 with the Rrp4-Myc proteins were detected by immunoblotting.

## DATA DEPOSITION

The raw RNA-seq data from this study have been submitted to the NCBI Gene Expression Omnibus (GEO) under accession GSE163106.

## SUPPLEMENTAL MATERIAL

Supplemental material is available for this article.

## Supplementary Material

Supplemental Material
