# Supplementary material for: A budding yeast model for human disease mutations in the EXOSC2 cap subunit of the RNA exosome complex
Source: RNA. 2021 Sep;27(9):1046–67. doi: 10.1261/rna.078618.120 (PMC8370739; doi:10.1261/rna.078618.120)
Supplement: Supplemental Material [file supp_27_9_1046__DC1.html]

A Budding Yeast Model for Human Disease Mutations in the EXOSC2 Cap Subunit of the RNA Exosome — A budding yeast model for human disease mutations in the EXOSC2 cap subunit of the RNA exosome complex — Supplemental Material 

# A budding yeast model for human disease mutations in the *EXOSC2* cap subunit of the RNA exosome complex

## Supplemental Material

- Supplemental\_Figure\_S1.pdf
- Supplemental\_Figure\_S2.pdf
- Supplemental\_Figure\_S3.pdf
- Supplemental\_Figure\_S4.pdf
- Supplemental\_Table\_S1.pdf
- Supplemental\_Table\_S2.pdf
- Supplemental\_Table\_S3.pdf
