# Supplementary material for: A budding yeast model for human disease mutations in the EXOSC2 cap subunit of the RNA exosome complex
Source: RNA. 2021 Sep;27(9):1046–67. doi: 10.1261/rna.078618.120 (PMC8370739; doi:10.1261/rna.078618.120)
Supplement: Supplemental Material [file supp_078618.120_Supplemental_Figure_S1.pdf]

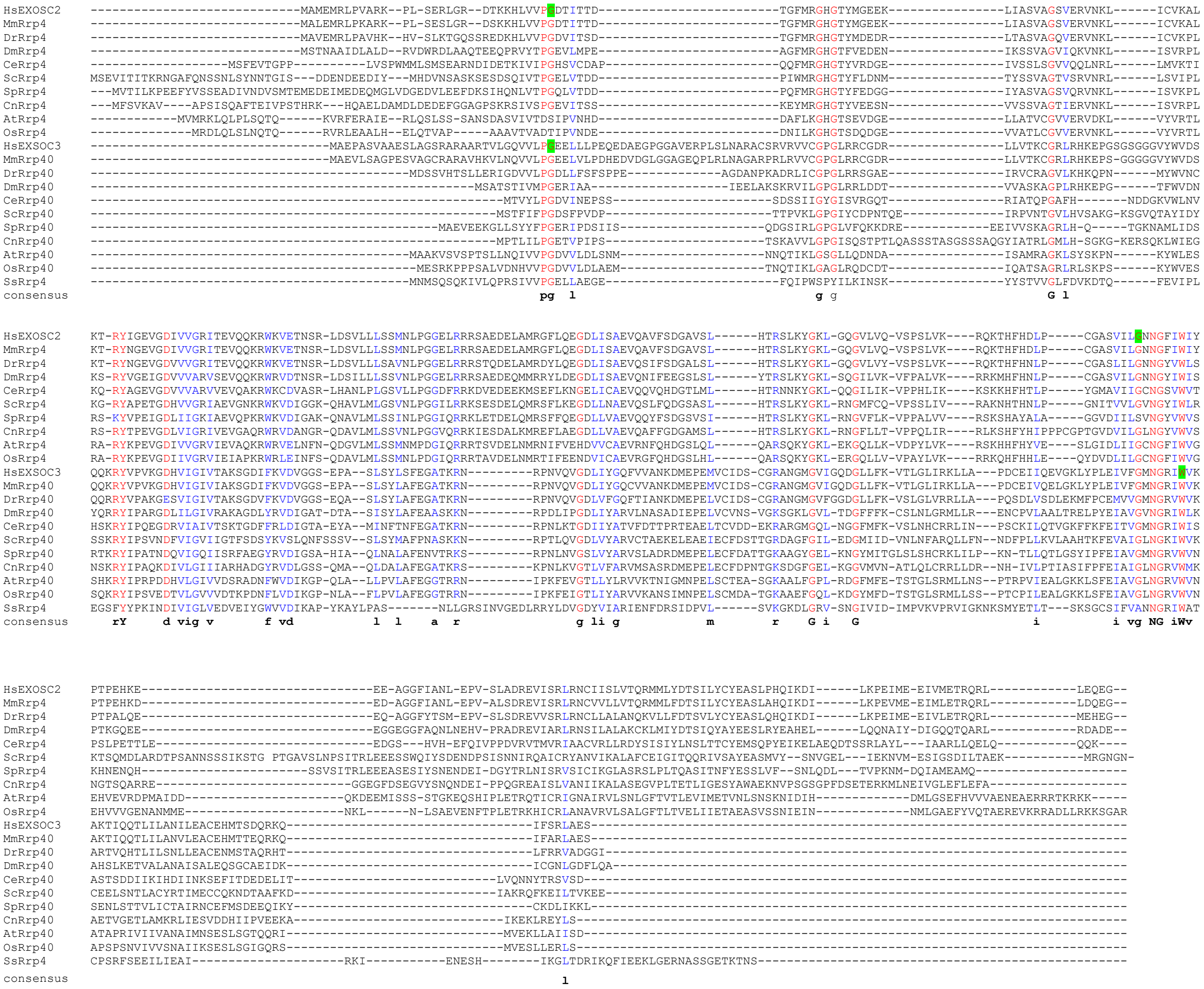

Supplementary Figure S1. Protein sequence alignment of human EXOSC2 and EXOSC3. Protein sequence alignment of the human RNA exosome cap subunits EXOSC2 and EXOSC3, and *S. cerevisiae* cap subunits Rrp4 and Rrp40, including other EXOSC2/Rrp4 and EXOSC3/Rrp40 orthologs. Conserved EXOSC2 amino acids substituted in patients with SHRF (Short stature, Hearing loss, Retinitis pigmentosa and distinctive Facies) syndrome (Di Donato et al. 2016) and EXOSC3 amino acids substituted in patients with PCH1b (pontocerebellar hypoplasia type 1b) disease (Wan et al. 2012) are highlighted in green. Identical residues (in red) and similar residues (in blue) are indicated. The different species aligned are as follows: Hs: *Homo sapiens*, Mm: *Mus musculus*, Dr: *Danio rerio*, Dm: *Drosophila melanogaster*, Ce: *Caenorhabditis elegans*, Sc: *Saccharomyces cerevisiae*, Sp: *Schizosaccharomyces pombe*, Cn: *Cryptococcus neoformans*, At: *Arabidopsis thaliana*, Os: *Oryza sativa*, Ss: *Sulfolobus solfataricus* (archaea).
