# Supplementary material for: A budding yeast model for human disease mutations in the EXOSC2 cap subunit of the RNA exosome complex
Source: RNA. 2021 Sep;27(9):1046–67. doi: 10.1261/rna.078618.120 (PMC8370739; doi:10.1261/rna.078618.120)
Supplement: Supplemental Material [file supp_078618.120_Supplemental_Figure_S2.pdf]

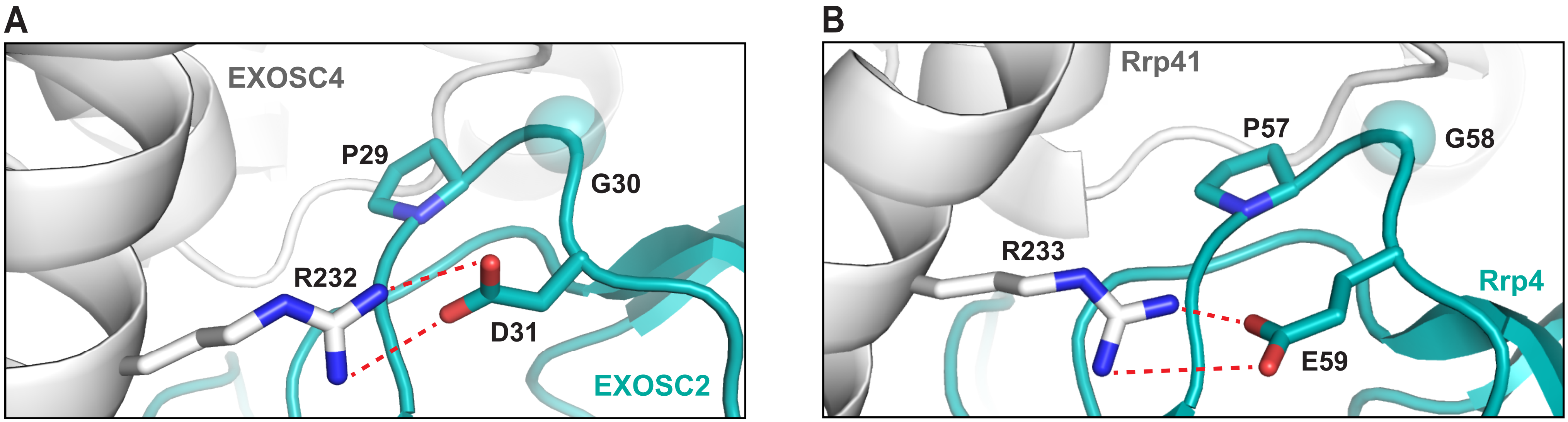

Supplementary Figure S2. Modeling of the human EXOSC2-EXOSC4 and yeast Rrp4-Rrp41 interface show structural conservation. (A) Zoomed-in representations of the interface between the human RNA exosome cap subunit EXOSC2 (teal blue) and core subunit EXOSC4 (light gray). EXOSC2 residue Gly30 (G30) facilitates a β turn that positions EXOSC2 residue Asp31 (D31) near an arginine in EXOSC4, Arg232 (R232). Structural modeling shows a salt bridge that forms between EXOSC2 D31 and EXOSC4 R232, represented by the red dashed lines. (B) Zoomed-in representation of the interface between the yeast RNA exosome cap subunit Rrp4 (teal blue) and core subunit Rrp41 (light gray). Rrp4 residue Gly58 (G58), which corresponds to EXOSC2 G30, facilitates a β turn that positions Rrp4 Glu59 (E59) near an arginine in the EXOSC4 yeast ortholog, Rrp41, Arg233 (R233). Structural modeling shows a salt bridge forms between Rrp4 E59 and Rrp41 R233, represented by the red dashed lines. Structural modeling in (A) was performed with the human RNA exosome structure (PDB 6D6R) (Weick et al. 2018) and in (B) with the yeast RNA exosome structure (PDB 6FSZ) (Schuller et al. 2018) using PyMOL (PyMOL).
