# Supplementary material for: A budding yeast model for human disease mutations in the EXOSC2 cap subunit of the RNA exosome complex
Source: RNA. 2021 Sep;27(9):1046–67. doi: 10.1261/rna.078618.120 (PMC8370739; doi:10.1261/rna.078618.120)
Supplement: Supplemental Material [file supp_078618.120_Supplemental_Figure_S3.pdf]

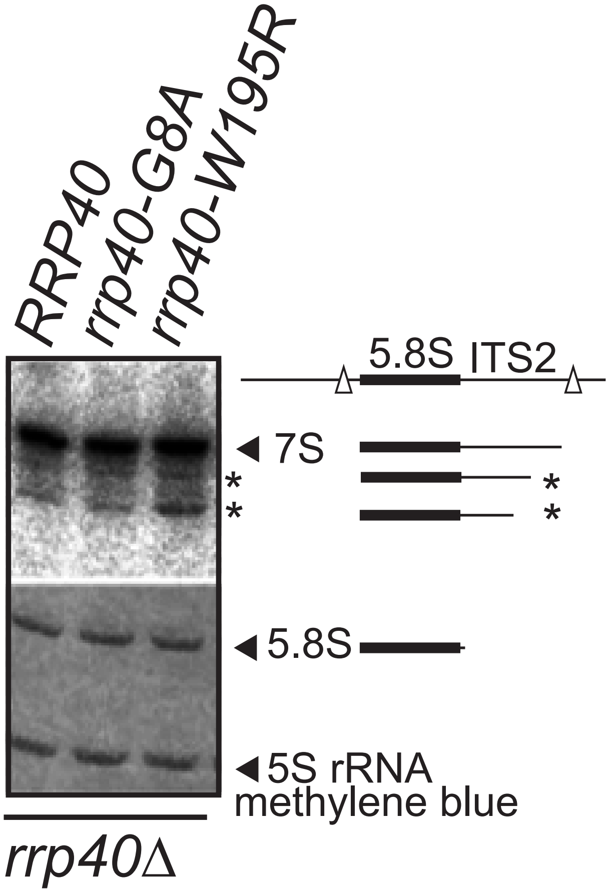

Supplementary Figure S3. Increased input signal levels for rRNA northern blot in Figure 5A emphasize previously observed accumulation of 5.8S precursors in *rrp40-W195R* cells. The lanes for the *RRP40, rrp40-G8A* and *rrp40-W195R* samples from the northern blot displayed in Figure 5A are shown here with the input signal levels increased. Total RNA from *RRP40*, *rrp40-G8A*, and *rrp40-W195R* cells grown at 37°C was analyzed by northern blotting with a 5.8S-ITS2 probe to detect 7S pre-rRNA. Mature 5.8S rRNA and 5S rRNA was detected by methylene blue staining as a loading control. The simplified schematics to the right illustrate the processing steps of 7S rRNA precursor following endonucleolytic cleavage from larger 27S precursor (indicated by white triangles). Accumulation of 5.8S precursors is evident in *rrp40-W195R* (labeled with asterisks).
