# Supplementary material for: A budding yeast model for human disease mutations in the EXOSC2 cap subunit of the RNA exosome complex
Source: RNA. 2021 Sep;27(9):1046–67. doi: 10.1261/rna.078618.120 (PMC8370739; doi:10.1261/rna.078618.120)
Supplement: Supplemental Material [file supp_078618.120_Supplemental_Figure_S4.pdf]

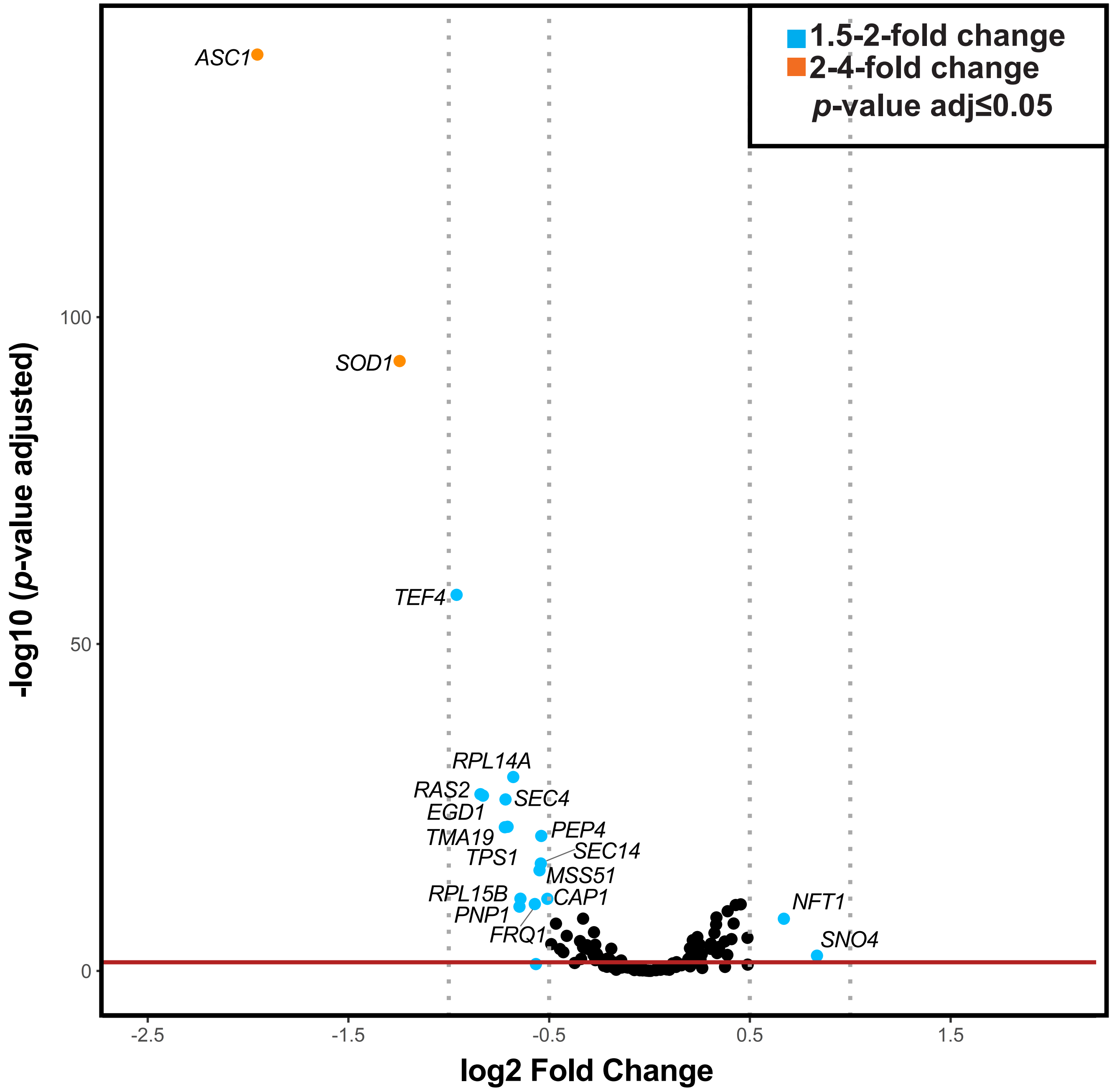

Supplementary Figure S4. Volcano plot of autophagy transcripts differentially expressed in the *rrp4-G226D* RNA-Seq. A total of 18 autophagy transcripts show ±1.5-fold change (p-value adjusted < 0.05) in *rrp4-G226D* cells compared to *RRP4* cells. Of those, 2 transcripts are increased 1.5-2 fold (*NFT1* and *SNO4* (blue)) and 16 transcripts are decreased 1.5-2-fold (*TEF4, RPL14A, RAS2, EGD1 SEC4, TPS1, TMA19, PEP4, SEC14, MSS51, RPL15B, CAP1, FRQ1, PNP1* (blue)) with two transcripts decreased > 2-fold (*ASC1* and *SOD1* (orange)).
