# Supplementary material for: A budding yeast model for human disease mutations in the EXOSC2 cap subunit of the RNA exosome complex
Source: RNA. 2021 Sep;27(9):1046–67. doi: 10.1261/rna.078618.120 (PMC8370739; doi:10.1261/rna.078618.120)
Supplement: Supplemental Material [file supp_078618.120_Supplemental_Table_S1.pdf]

Yeast Strains and Plasmids

Strain/Plasmid	Description	Source
rrp4Δ (yAV1103)	*MATα, ura3Δ0, leu2Δ0, his3Δ1, lys2Δ0, rrp4Δ::NEO,* [*RRP4,URA3*]	(LOSH 2018)
rrp40Δ (yAV1107)	*MATa, ura3Δ0, leu2Δ0, his3Δ1, rrp40Δ::NEO,* [*RRP40,URA3*]	(SCHAEFFER *et al.* 2009)
rrp4Δ mpp6Δ(ACY2471)	*MATα, ura3Δ0, leu2Δ0, his3Δ1, lys2Δ0, rrp4Δ::NEO, mpp6Δ::natMX4,* [*RRP4, URA3*]	This study
rrp4Δ rrp47Δ (ACY2474)	*MATα, ura3Δ0, leu2Δ0, his3Δ1, lys2Δ0, rrp4Δ::NEO, rrp47Δ::natMX4,* [*RRP4, URA3*]	This study
rrp4Δ rrp6Δ (ACY2478)	*MATα, ura3Δ0, leu2Δ0, his3Δ1, lys2Δ0, rrp4Δ::NEO, rrp6Δ::natMX4,* [*RRP4, URA3*]	This study
rrp40Δ mpp6Δ (ACY2638)	*MATa, ura3Δ0, leu2Δ0, his3Δ1, rrp40Δ::NEO, mpp6Δ::natMX4,* [*RRP40, URA3*]	This study
rrp40Δ rrp47Δ (ACY2462)	*MATa, ura3Δ0, leu2Δ0, his3Δ1, rrp40Δ::NEO, rrp47Δ::natMX4,* [*RRP40, URA3*]	This study
rrp40Δ rrp6Δ (ACY2466)	*MATa, ura3Δ0, leu2Δ0, his3Δ1, rrp40Δ::NEO, rrp6Δ::natMX4,* [*RRP40, URA3*]	This study
RRP43-TAP (ACY2788)	*MATa, ura3Δ0, leu2Δ0, his3Δ1, met15Δ0, RRP43-TAP:HIS3MX6*	(GHAEMMAGHAMI *et al.* 2009)
RRP43-TAP rrp4Δ (ACY2803)	*MATa, ura3Δ0, leu2Δ0, his3Δ1, met15Δ0, RRP43-TAP:HIS3MX6, rrp4Δ::neoMX,* [*RRP4, URA3*]	This study
pRS315	*CEN6, LEU2, amp*^{R}	(SIKORSKI AND HIETER 1989)
pRS313	*CEN6, HIS3, amp*^{R}	(SIKORSKI AND HIETER 1989)
pAC3161	*RRP40-2xMyc* in pRS315, *CEN6, LEU2, amp*^{R}	(FASKEN *et al.* 2017)
pAC3162	*rrp40-G8A-2xMyc* in pRS315, *CEN6, LEU2, amp*^{R}	(FASKEN *et al.* 2017)
pAC3259	*rrp40-W195R-2xMyc* in pRS315, *CEN6, LEU2, amp*^{R}	(FASKEN *et al.* 2017)
pAC3652	*RRP40-Native 3'UTR* in pRS315, *CEN6, LEU2, amp*^{R}	This study
pAC3655	*rrp40-W195R-Native 3'UTR* in pRS315, *CEN6, LEU2, amp*^{R}	This study
pAC3474	*RRP4-2xMyc* in pRS315, *CEN6, LEU2, amp*^{R}	This study
pAC3476	*rrp4-G58V-2xMyc* in pRS315, *CEN6, LEU2, amp*^{R}	This study
pAC3477	*rrp4-G226D-2xMyc* in pRS315, *CEN6, LEU2, amp*^{R}	This study
pAC3656	*RRP4-Native 3'UTR* in pRS315, *CEN6, LEU2, amp*^{R}	This study
pAC3659	*rrp4-G226D-Native 3'UTR* in pRS315, *CEN6, LEU2, amp*^{R}	This study
pAC3669	*RRP4-2xMyc-Native 3'UTR* in pRS315, *CEN6, LEU2, amp*^{R}	This study
pAC3670	*rrp4-G58V-2xMyc-Native 3'UTR* in pRS315, *CEN6, LEU2, amp*^{R}	This study
pAC3672	*rrp4-G226D-2xMyc-Native 3'UTR* in pRS315, *CEN6, LEU2, amp*^{R}	This study
pAC2897	*MTR4, 2μ, URA3, amp*^{R}	(FASKEN *et al.* 2011)
pAC3713	*MTR4, RRP40, CEN6, URA3, amp*^{R}	This study
pAC3714	*MTR4, RRP4, CEN6, URA3, amp*^{R}	This study
pAC3719	*MTR4-2xFLAG-Native 3'UTR* in pRS313, *CEN6, HIS3, amp*^{R}	This study
pAC4096	*MTR4-Native 3'UTR* in pRS313, *CEN6, HIS3, amp*^{R}	This study
pAC4099	*mtr4-F7A-F10A-Native 3'UTR* in pRS313, *CEN6, HIS3, amp*^{R}	This study

Table S1. *S. cerevisiae* Strains and Plasmids used in this study.
