# Supplementary material for: A budding yeast model for human disease mutations in the EXOSC2 cap subunit of the RNA exosome complex
Source: RNA. 2021 Sep;27(9):1046–67. doi: 10.1261/rna.078618.120 (PMC8370739; doi:10.1261/rna.078618.120)
Supplement: Supplemental Material [file supp_078618.120_Supplemental_Table_S2.pdf]

DNA Oligonucleotides used for Quantitative RT-PCR

**Description**	**Sequence (5'-3')**	**Name**
pre-*U4* snRNA Fwd	ATCCTTATGCACGGGAAATACG	AC5722
pre-*U4* snRNA Rev	AAAGAATGAATATCGGTAATG	AC5723
U14 snoRNA (snR128) Fwd	GATCACGGTGATGAAAGACTGG	AC5397
U14 snoRNA (snR128) Rev	CTACAGTATACGATCACTCAGACATCCTA	AC5398
snR44 snoRNA Fwd	GCATTTCCACATGGGATTAAA	AC6270
snR44 snoRNA Rev	ATGGTGTGATCGGGCAGTAT	AC6272
TLC1 ncRNA Fwd	AAGGCAAGGGTGTCCTTTCT	AC6420
TLC1 ncRNA Rev	TTCCGCTTGGAAAATAATGC	AC6421
pre-*TLC1* ncRNA Fwd	GTATTGTAGAAATCGCGCGTAC	AC7593
pre-*TLC1* ncRNA Rev	CCGCCTATCCTCGTCATGAAC	AC7594
RPS3 mRNA Fwd	TCCAACCAAGACCGAAGTTATC	AC9226
RPS3 mRNA Rev	GTACCTGGAGCGTACTTGAATC	AC9227
RPL15A mRNA Fwd	CCAGACAAGGCTAGAAGATTGG	AC9309
RPL15A mRNA Rev	CCGTAAGTAGCACCCTTTGG	AC9308
INO1 mRNA Fwd	TTGGACTGCAAATACTGAGAGG	AC9303
INO1 mRNA Rev	AAGATCGTGGAAGGAGCAATC	AC9302
PTH4 mRNA Fwd	ACTGTGCTTGGATTCCTCAG	AC9248
PTH4 mRNA Rev	CTATAGAATCGCTGCCCTTAGC	AC9249
HXK2 mRNA Fwd	TACTGGTGTCAATGGTGCTTAC	AC9307
HXK2 mRNA Rev	TTGGAGCAGATGGTGGAATG	AC9306
TDH1 mRNA Fwd	GGTAGATACAAGGGTACTGTTTCC	AC9230
TDH1 mRNA Rev	TGAGCGGTGTCCAATTCC	AC9232
CUT501 ncRNA Fwd	GGTTCAACGTTGCAGGATCT	AC9254
CUT501 ncRNA Rev	GCTAGCACCTGTTGCTGTAAT	AC9255
CUT770 ncRNA Fwd	AAACAACCCGCTAGTGTGAC	AC9262
CUT770 ncRNA Rev	AGAGCAACTCACTGCAAAGG	AC9263
CUT896 ncRNA Fwd	ATCAGCAGGTGTCATGTTACAG	AC9256
CUT896 ncRNA Rev	CCCAGAGGCAAAGATGTTAAGT	AC9257
NRD1 mRNA Fwd	CAAGCAGAGGTCGAAACAAATC	AC9244
NRD1 mRNA Rev	GCTGGATCTGTGGAAGTCAA	AC9245
NAB3 mRNA Fwd	ACAGTTCGGTAGGCTCAGATAG	AC9246
NAB3 mRNA Rev	GGCGAAGTTCGACCTCTTTATC	AC9247
ALG9 mRNA Fwd	CACGGATAGTGGCTTTGGTGAACAATTAC	AC5067
ALG9 mRNA Rev	TATGATTATCTGGCAGCAGGAAAGAACTTGGG	AC5068

Table S2. DNA Oligonucleotides employed for RT-qPCR
