# Supplementary material for: A budding yeast model for human disease mutations in the EXOSC2 cap subunit of the RNA exosome complex
Source: RNA. 2021 Sep;27(9):1046–67. doi: 10.1261/rna.078618.120 (PMC8370739; doi:10.1261/rna.078618.120)
Supplement: Supplemental Material [file supp_078618.120_Supplemental_Table_S3.pdf]

Exosomopathy amino acid substitution	*Hs* EXOSC2 G30V	*Hs* EXOSC2 G198D	*Sc* Rrp4 G58V	*Sc* Rrp4 G226D
**Missense 3D* (Phyre2)**	Neutral	Damaging	Neutral	Damaging
**PolyPhen-2** (HumDiv)**	Possibly Damaging (Score: 1.000)	Probably Damaging (Score: 1.000)	Probably Damaging (Score: 1.000)	Probably Damaging (Score: 1.000)
**Provean†**	Deleterious (-7.938)	Deletrious (-6.35)	Deletrious (-8.981)	Deletrious (-6.517)
**SNAP2‡**	Effect (Score: 91)	Effect (Score: 94)	Effect (Score: 63	Effect (Score: 92)

***Missense 3D** part of tool suite **Phyre2** (**Protein H**omology/analog**Y Re**cognition Engine) v2.0; predicts structural changes introducted by an amino acid substitution through three-dimensional protein modeling.

****PolyPhen-2** (**Poly**morphism **Phen**otyping) v2 HumDiv trained model; Naïve Bayes posterior probability that mutation is damaging and qualitative classification as benign, possibly damaging, or probably damaging based on 5%/10% false positive rate (FPR) thresholds (FPR, the chance the mutation is classified as damaging when it is non-damaging).

†**PROVEAN** (**Pro**tein **V**ariation **E**ffect **An**alyzer) v1.1; delta alignment scores equal to or below predefined threshold (-2.5), protein variant predicited to have “deleterious” effect on function.

‡**SNAP2** predicted score for functional effects of mutations; scores range from -100 strong neutral prediction to +100 strong effect prediction.

Table S3. Summary of *in silico* predictions for pathogenic amino acid substitutions in EXOSC2 and Rrp4.
